# Buspirone alleviates anxiety, depression, and colitis; and modulates gut microbiota in mice

**DOI:** 10.1038/s41598-021-85681-w

**Published:** 2021-03-17

**Authors:** Jeon-Kyung Kim, Sang-Kap Han, Min-Kyung Joo, Dong-Hyun Kim

**Affiliations:** Neurobiota Research Center, College of Pharmacy, Kyung Hee University, 26, Kyungheedae-ro, Dongdaemun-gu, Seoul, 02447 South Korea

**Keywords:** Drug discovery, Microbiology, Neuroscience

## Abstract

Gut microbiota regulate the neurodevelopmental processes and brain functions through the regulation of the microbiota–gut interaction and gut–brain communication. Buspirone, an agonist for serotonin 5-HT1A receptors, is used for the treatment of anxiety/depression. Therefore, to understand the gut microbiota-mediated mechanism of buspirone on anxiety/depression, we examined its effect on the immobilization stress (IS) or *Escherichia coli* K1 (EC)-induced anxiety/depression in mice. Oral or intraperitoneal administration of buspirone significantly suppressed stressor-induced anxiety/depression-like behaviors in the elevated plus maze, light/dark transition, tail suspension, and forced swimming tasks. Their treatments also reduced TNF-α expression and NF-κB^+^/Iba1^+^ cell population in the hippocampus and myeloperoxidase activity and NF-κB^+^/CD11c^+^ cell population in the colon. Buspirone treatments partially restored IS- or EC-induced gut microbiota perturbation such as β-diversity to those of normal control mice: they reduced the IS- or EC-induced gut Proteobacteria population. In particular, the anxiolytic activity of buspirone was positively correlated with the populations of Bacteroides and PAC001066_g in EC- or IS-exposed mice, while the populations of Lachnospiraceae, KE159660_g, LLKB_g, Helicobacter, and PAC001228_g were negatively correlated. The anti-depressant effect of buspirone was positively correlated with the Roseburia population. The fecal microbiota transplantations from buspirone-treated mice with IS-induced anxiety/depression or normal control mice suppressed IS-induced anxiety/depression-like behaviors and reduced hippocampal NF-κB^+^/Iba1^+^ and colonic NF-κB^+^/CD11c^+^ cell populations in the transplanted mice. Furthermore, they modified IS-induced perturbation of gut microbiota composition, particularly Proteobacteria, in the transplanted mice. In conclusion, buspirone alleviates IS as well as EC-induced anxiety/depression and colitis. It also suppresses associated neuroinflammation and modulates gut microbiota. Future studies can help to explain the relationship, if any, in the central and peripheral effects of buspirone.

## Introduction

Anxiety/depression, the most prevalent mental disorder, are raised by exposure to stressors such as immobilization, social defeat, forced swimming, and pathogen infection^1,2^. Patients with anxiety disorder progress to the depressive disorder that is a common illness worldwide^2^. Exposure to stressors such as immobilization stress (IS) and social defeat stimulates the secretion of adrenaline and glucocorticoids in the adrenal gland via the hypothalamic–pituitary–adrenal (HPA) axis and tumor necrosis factor (TNF)-α and interleukin (IL)-6 in immune cells, leading to the outbreak of anxiety/depression and gut inflammation^3,4^. The excessive stimulation of these glucocorticoids and proinflammatory cytokines causes gut inflammation and microbiota perturbation by the activation of innate and adaptive immunities in the gastrointestinal tract^5,6^. Gut microbiota perturbation inversely collapses the immune and central nervous systems via the microbiota–gut interaction and gut–brain communication (MGB), leading to the outbreak of anxiety/depression^7,8^. For example, the overgrowth of *Klebsiella oxytoca* by oral gavage of antibiotics or oral gavage of *Escherichia coli* causes anxiety and depression with colitis in mice^9,10^. Anti-inflammatory therapy in patients with inflammatory bowel disease (IBD) alleviates psychiatric disorder^11,12^.

Many medications, such as benzodiazepines and selective serotonin reuptake inhibitors, (SSRIs) are used for the treatment of anxiety/depression^13,14^. Of these, buspirone is known to be a partial agonist for the serotonin 5-HT1A receptors as well as an antagonist for the dopamine D2 autoreceptors. However, low-dose buspirone activates only 5-HT1A receptors. Its anxiolytic and anti-depressant effects are produced by activating 5-HT1A autoreceptors and 5-HT1A heteroreceptors, respectively^15,16^. Anti-depressant drugs alleviate colitis in mice with colitis^17^. However, the effects of buspirone on stressor-induced colitis and gut dysbiosis have not been studied.

Therefore, to clarify the action mechanism of buspirone, we examined the gut microbiota-mediated effects of buspirone on HPA axis-triggering IS- or MGB-triggering *Escherichia coli* K1 stress (EC)-induced anxiety/depression, colitis, and gut microbiota perturbation in mice.

## Results

### Effect of orally gavaged or intraperitoneally injected buspirone on IS-induced depression in mice

**Figure 1:**
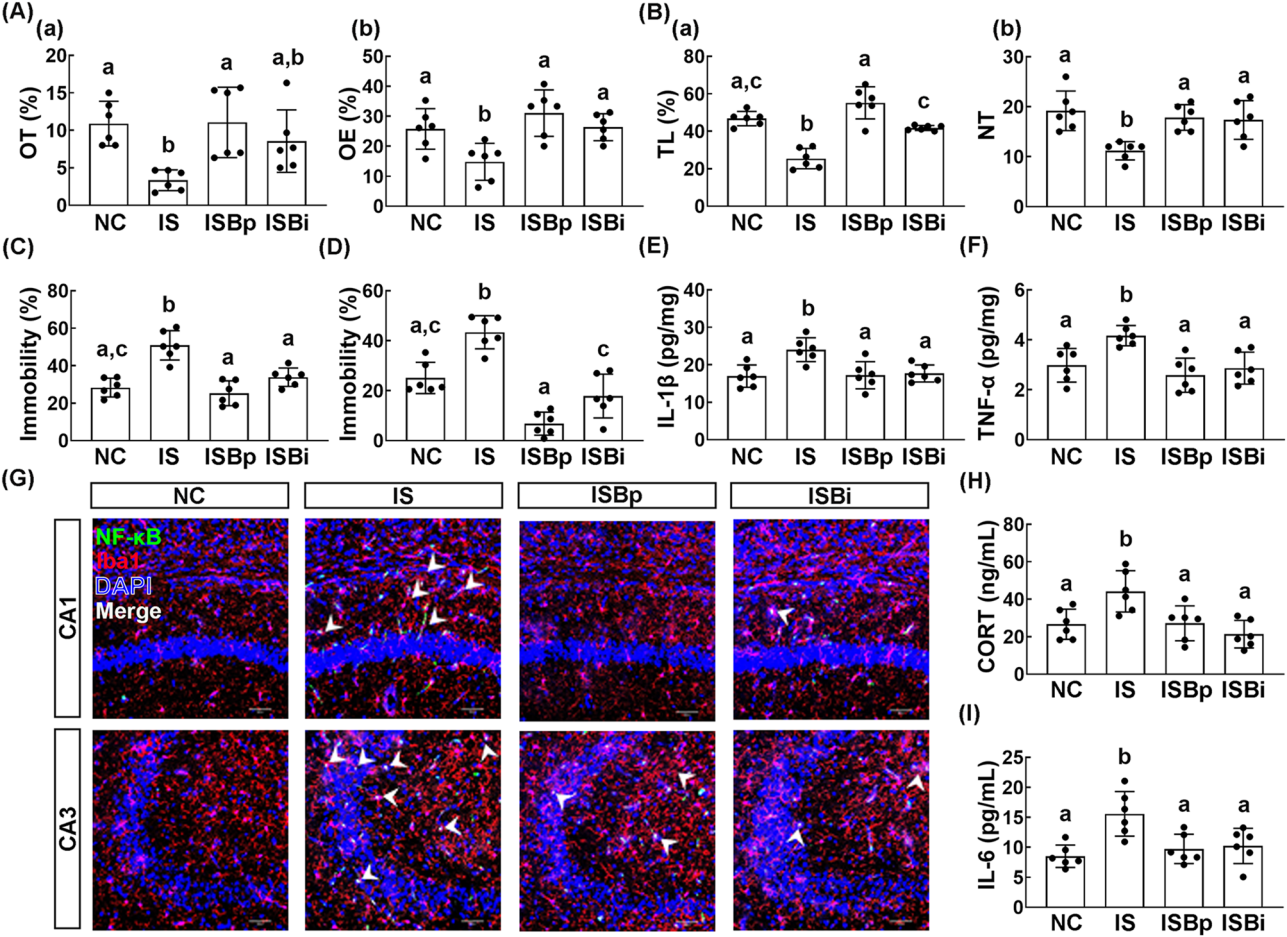
Orally gavage or intraperitoneal injection of buspirone alleviated immobilization stress (IS)-induced anxiety/depression in mice. Effects on IS-induced anxiety/depression-like behaviors in EPM ((**A**) a: time spent in the open arm [OT]; b: open arm entries [OE]), LDT ((**B**) a: time spent in the light box [TL]; b: number of transition into the light box entry [NT]), TST (**C**), and FST (**D**). Effects on IL-1β (**E**) and TNF-α expression (**F**) and NF-κB^+^/Iba1^+^ cell population (**G**) in the hippocampus. Effect on corticosterone (CORT, **H**) and IL-6 levels (**I**) in the blood. Buspirone was orally gavaged (IBPp, 5 mg/kg/day) or intraperitoneally injected (IBpi, 1 mg/kg/day) daily for 5 days from the next day after the final exposure to immobilization stress (IS). IS and NC (normal control mice) were orally treated with vehicle (saline) instead of buspirone. Data values were indicated as mean ± SD (n = 6). Same letters are not significantly different (*p* < 0.05).

First, we examined the effect of orally gavaged or intraperitoneally injected buspirone on the IS-induced anxiety/depression in mice. Exposure to IS increased the anxiety-/depression-like behaviors: it decreased the time spent in the open arm (OT) in the elevated plus maze (EPM) task and time spent in the light box (TL) in the light/dark transition (LDT) task and increased immobility times in the tail suspension test (TST) and forced swimming test (FST) (Fig. 1A–D). However, oral administration of buspirone significantly reduced IS-induced anxiety-/depression-like behaviors and suppressed IL-1β and TNF-α expression and NF-κB^+^/Iba1^+^ cell population in the hippocampus (Fig. 1A–G, Supplementary Fig. S1A). Intraperitoneal injection of buspirone also suppressed IS-induced anxiety-/depression-like behaviors in the EPM and LDT tasks, TST, and FST (Fig. 1A–D). Intraperitoneal injection of buspirone suppressed the IS-induced TNF-α and IL-1β expression and NF-κB^+^/Iba1^+^ cell population in the hippocampus (Fig. 1E–G). Furthermore, oral gavage and intraperitoneal injection of buspirone suppressed corticosterone and IL-6 levels in the blood (Fig. 1H,I).

**Figure 2:**
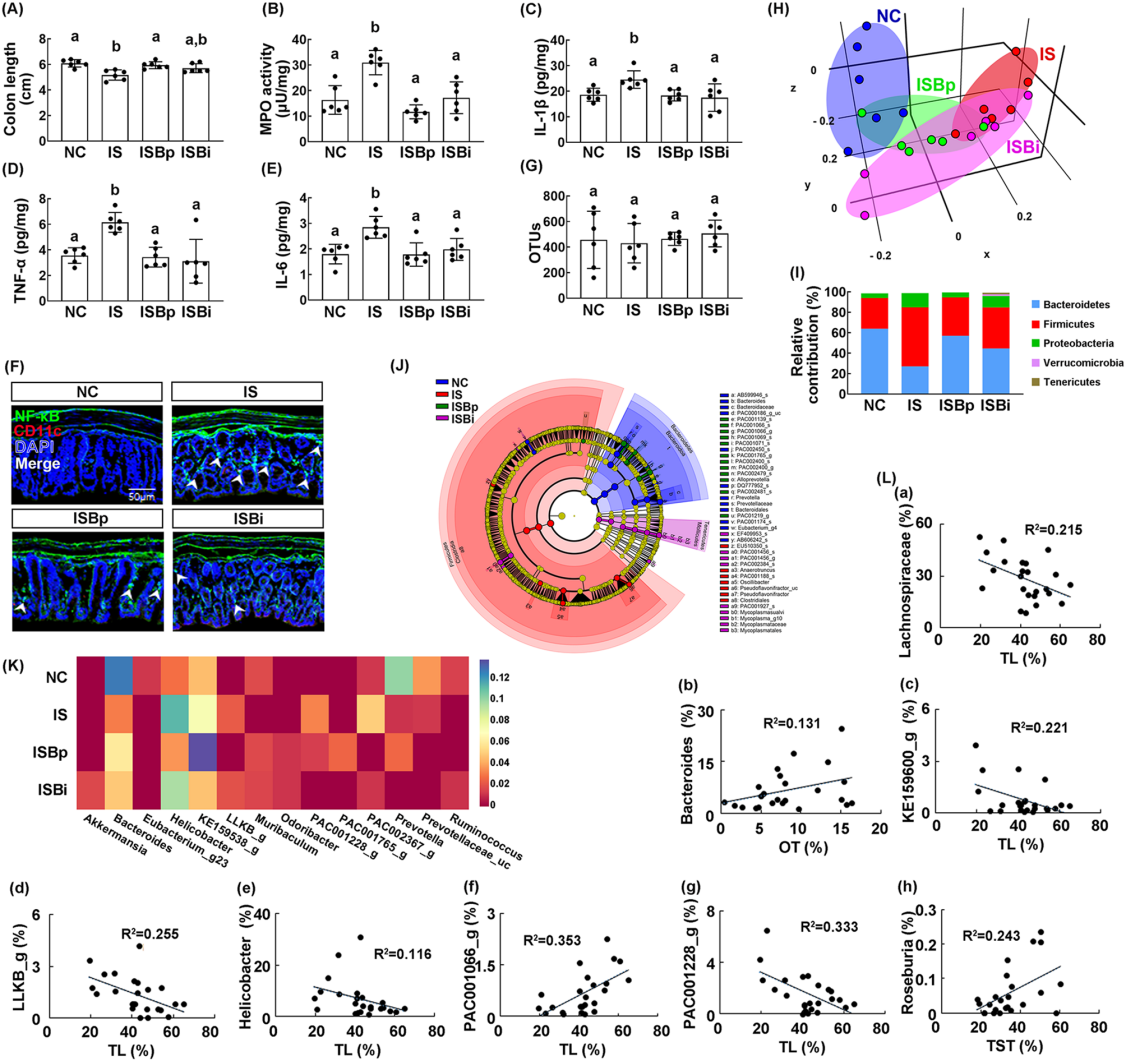
Orally gavage or intraperitoneal injection of buspirone alleviated immobilization stress (IS)-induced colitis and gut microbiota pertubation in mice. Effects on IS-induced colon shortening (**A**), myeloperoxidase (MPO) activity (**B**), IL-1β (**C**), TNF-α (**D**), and IL-6 expression (**E**), and NF-κB^+^/CD11c^+^ cell population (**F**) in the colon. Effects on the composition of gut microbiota: estimated operational taxonomic units (OTUs) (**G**), principal coordinate analysis (PCoA) plot (**H**) based on generalized UniFrac analysis. Composition of gut miciobiota analyzed in phylum level (**I**). (**J**) Cladogram was generated by LEfSE indicating significant differences in gut microbial abundances among untreated control (NC, blue), IS-treated (IS, red), orally buspirone-treated (ISBp, green), and intraperitoneally buspirone-treated (ISBi, purple) groups. Yellow nodes represent species with no significant difference. The threshold logarithmic score set at 3.5 in the family level and ranked. (**K**) Effects on gut microbiota composition at the genus level, expressed as heat map. (**L**) The correlation between gut microbiota composition (**L**: a, family level; b–h, genus level) and anxiety-/depression-like behaviors, assessed in the EPM (time spent in the open arm [OT]), LDT (time spent in the light box [TL]), and TST (immobility time). Buspirone was orally gavaged (IBPp, 5 mg/kg/day) or intraperitoneally injected (IBpi, 1 mg/kg/day) daily for 5 days from the next day after the final exposure to IS. IS and NC (normal control mic) were orally treated with vehicle (saline) instead of buspirone. (**H**) and (**K**) were created in the the free Plotly Make Chart program (https://chart-studio.plotly.com/create/#/). (**J**) was created by using LEfSe analysis tool from the galaxy site (https://huttenhower.sph.harvard.edu/galaxy/). Data values were indicated as mean ± SD (n = 6). Same letters are not significantly different (*p* < 0.05).

Exposure to IS significantly induced colon shortening, myeloperoxidase activity, IL-1β, IL-6, and TNF-α expression, and NF-κB^+^/CD11c^+^ cell population in the colon, resulting in colitis (Fig. 2A–F, Supplementary Fig. S1B). However, oral gavage and intraperitoneal injection of buspirone alleviated IS-induced colitis: it suppressed myeloperoxidase activity, IL-1β, IL-6, and TNF-α expression, and NF-κB^+^/CD11c^+^ cell population in the colon.

Exposure to IS also perturbed gut microbiota composition in mice: it changed the β-diversity while the α-diversity was not affected (Fig. 2G,H). Furthermore, it increased Proteobacteria and Firmicutes populations and reduced Bacteroidetes population (Fig. 2I). However, oral gavage and intraperitoneal injection of buspirone partially restored IS-shifted β-diversity in the gut microbiota to that of control mice, while the α-diversity (OTUs) was not affected (Fig. 2G–J). They reduced IS-induced Proteobacteria and Firmicutes populations and increased Bacteroidetes and Verrucomicrobiota populations (Fig. 2I,J, Supplementary Fig. S2). In particular, oral gavage of buspirone increased IS-suppressed Bacteroidaceae and Muribaculaceae populations at the family level and PAC000186_g, PAC000198_g, PAC001068_g, PAC001765_g populations at the genus level and PAC001064_s and PAC001065_s populations at the species level and reduced IS-induced Helicobacteriaceae and Ruminococcaceae populations at the family, Helicobacter, LLKB_g, Oscillibacter, PAC000664_g, Pseudoflavonifractor populations at the genus level, and *Helicobacter rodentium* group at the species level (Supplementary Tables S1–S3). Intraperitoneal injection of buspirone increased IS-suppressed Akkermansiaceae and Rikenellaceae populations at the family level, Bacteroides populations at the genus level, and AB599946_s group population at the species level and reduced IS-induced Lachnospiraceae and Ruminococcaceae populations at the family, Oscillibacter, PAC000664_g, and PAC001228_g populations at the genus level, and KE159538_g_uc at the species level (Supplementary Tables S1–S3). Next, we visualized the gut microbiota composition of mice treated with and without buspirone as a heat map at the genus level (Fig. 2K, Supplementary Table S2). Oral gavage or intraperitoneal injection of buspirone reduced IS exposure-induced Bacteroides population, while IS-suppressed Muribaculum population increased. Oral gavage of buspirone significantly reduced IS-induced Helicobacter and LLKB_g populations. Intraperitoneal injection of buspirone significantly increased IS-suppressed Rumonococcus population and decreased IS-induced PAC001228_g and PAC002367_g populations. Oral gavage or intraperitoneal injection of buspirone increased Odoribacter population. To understand what kinds of gut microbiota are associated with the therapeutic effects of buspirone in mice with IS-induced anxiety/depression, we analyzed the correlation between anxiety-/depression-like behaviors and gut microbiota in mice treated with and without buspirone (Fig. 2L). Lachnospiraceae (R = − 0.464, R^2^ = 0.215, p = 0.022), KE159600_g (R = − 0.470, R^2^ = 0.221, p = 0.021), LLKB_g (R = − 0.505, R^2^ = 0.255, p = 0.012), Helicobacter (R = − 0.341, R^2^ = 0.116, p = 0.103) and PAC001228_g populations (R = − 0.577, R^2^ = 0.333, p = 0.003) showed the negative correlation with the anxiety-like behavior (TL) in the LDT task, while Bacteroides population (R = 0.361, R^2^ = 0.131, p = 0.083) showed the positive correlation with the anxiety-like behavior (OT) in the EPM task. Roseburia population (R = 0.493, R^2^ = 0.243, p = 0.014) had the positive correlation with the depression-like behavior in the TST.

### Effect of orally gavaged or interaperitoneally injected buspirone on EC-induced depression and colitis in mice

**Figure 3:**
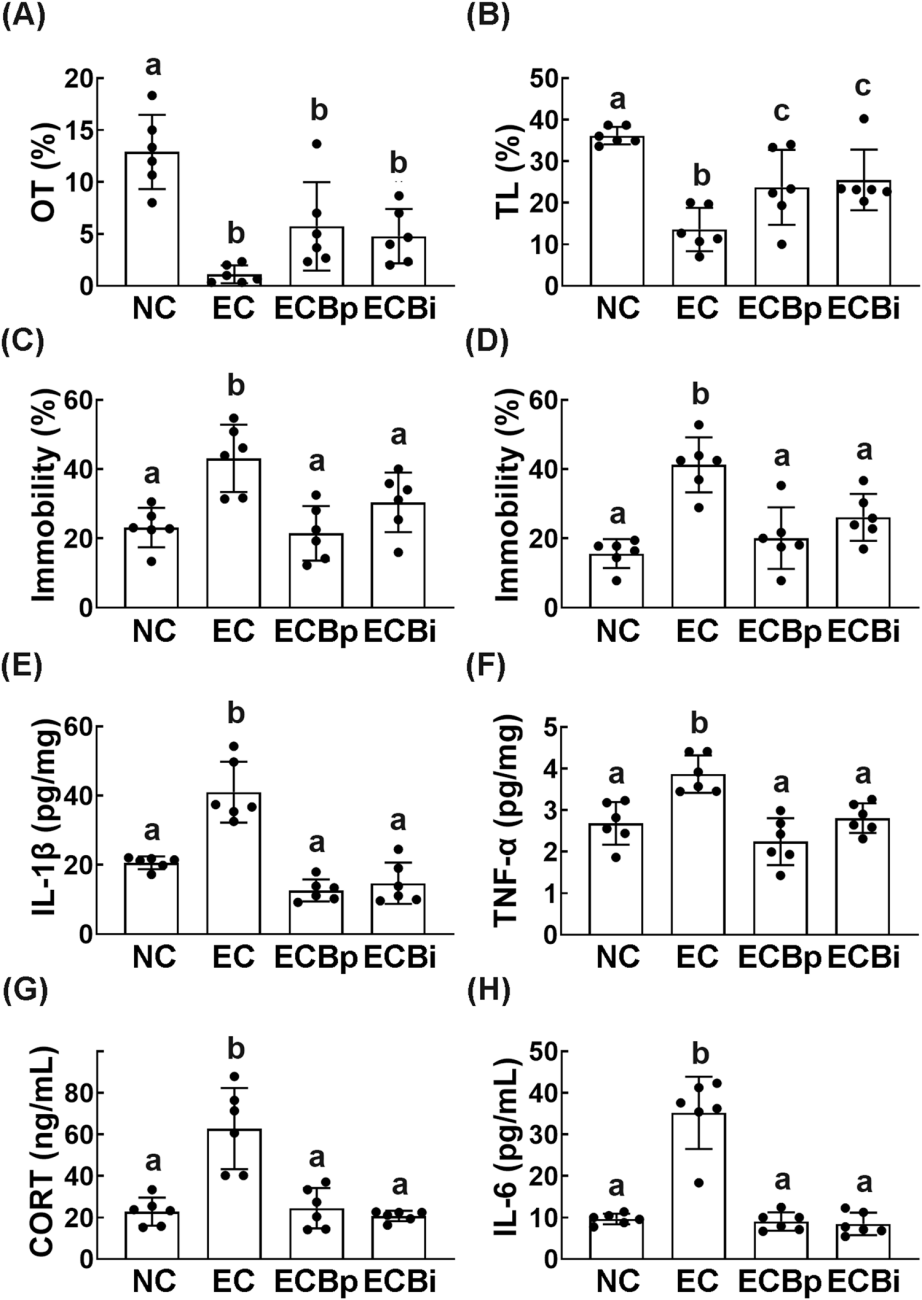
Orally gavage or intraperitoneal injection of buspirone alleviated *Escherichia coli* (EC)-induced anxiety/depression in mice. Effects on EC-induced anxiety/depression-like behaviors in EPM (**A**, OT), LDT (**B**, TL), TST (**C**), and FST (**D**). Effects on IL-1β (**E**) and TNF-α expression (**F**) in the hippocampus. Effect on corticosterone (CORT, **G**) and IL-6 levels (**H**) in the blood. Buspirone was orally gavaged (EBPp, 5 mg/kg/day) or interaperitoneally injected (EBpi, 1 mg/kg/day) daily for 5 days from the next day after the final exposure to EC. EC and NC (normal control mice) was orally treated with vehicle (saline) instead of buspirone. Data values were indicated as mean ± SD (n = 6). Same letters are not significantly different (*p* < 0.05).

We also examined the effect of orally gavaged or intraperitoneal injected buspirone on EC*-*induced anxiety/depression in mice. Exposure to EC decreased the OT in the EPM task and TL in the LDT task and increased the immobility time in the TST and FST, resulting in anxiety/depression (Fig. 3A–D, Supplementary Fig. S3). However, orally gavaged or intraperitoneally injected buspirone significantly reduced EC*-*induced anxiety-/depression-like behaviors, suppressed EC*-*induced TNF-α and IL-1β expression in the hippocampus (Fig. 3A–F, Supplementary Fig. S3A,B). Buspirone also suppressed EC*-*induced corticosterone and IL-6 levels in the blood (Fig. 3G,H).

**Figure 4:**
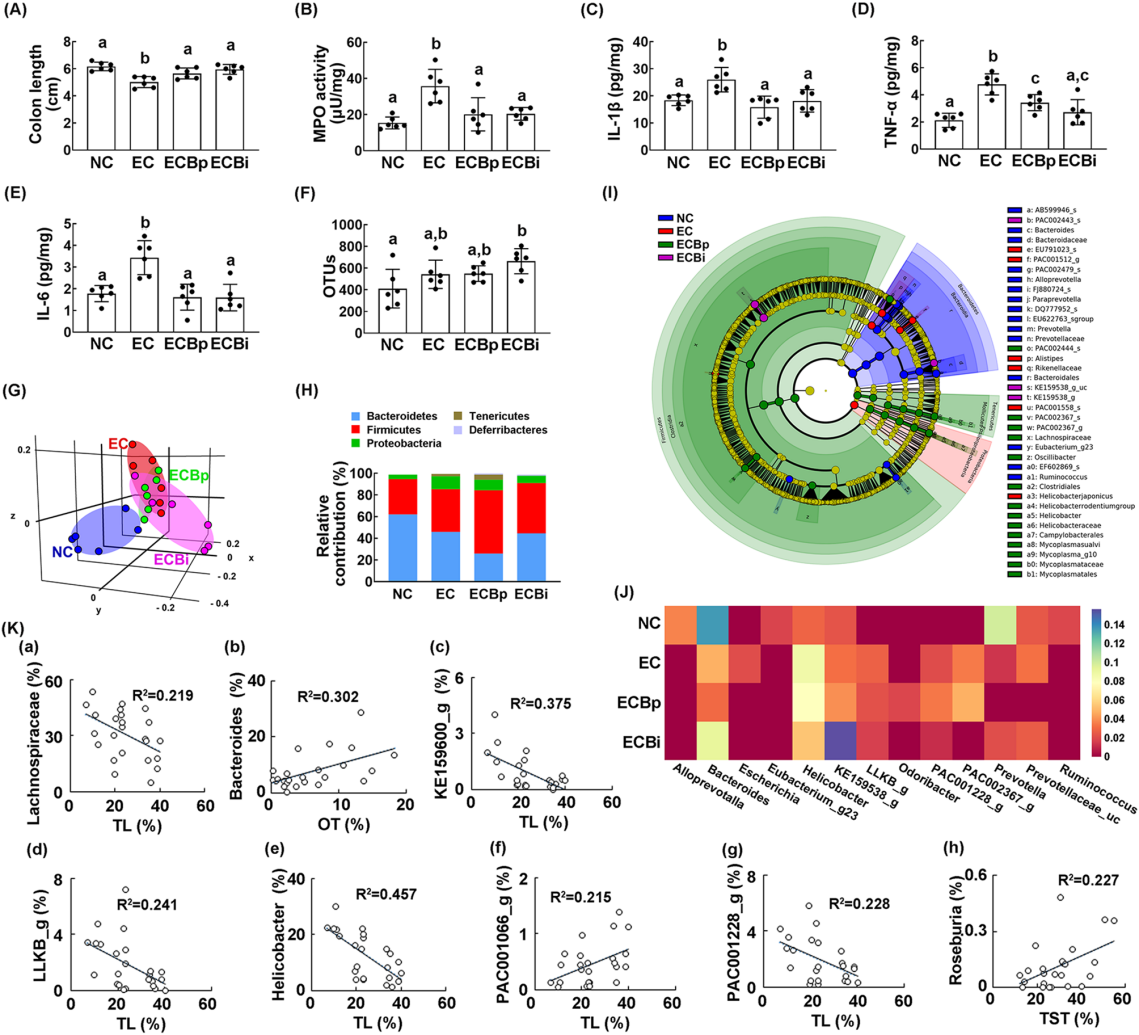
Orally gavage or intraperitoneal injection of buspirone alleviated *Escherichia coli* (EC)-induced colitis and gut microbiota pertubation in mice. Effects on IS-induced colon shortening (**A**), myeloperoxidase (MPO) activity (**B**), and IL-1β (**C**) and TNF-α (**D**) expression (**E**) in the colon. Effects on the composition of gut microbiota: OTUs (**F**), and principal coordinate analysis (PCoA) plot based on generalized UniFrac analysis (**G**). Composition of gut miciobiota were analyzed in phylum level (**H**). Cladogram was generated by LEfSE indicating (**I**) significant differences in gut microbial abundances among normal control (NC, blue), EC-treated (EC, red), orally buspirone-treated (ECBp, green), and intraperitoneally buspirone-treated (ECBi, purple) groups. (**J**) Effects on gut microbiota composition at the genus level, expressed as heat map. (**K**) The correlation between gut microbiota composition (a, family level; b–h, genus level) and anxiety-/depression-like behaviors, assessed in the EPM (time spent in the open arm [OT]), LDT (time spent in the light box [TL]), and TST (immobility time). Buspirone was orally gavaged (ECBp, 5 mg/kg/day) or intraperitoneally injected (ECBi, 1 mg/kg/day) daily for 5 days from the next day after the final exposure to EC. NC and NC (normal control mice) were orally treated with vehicle (saline) instead of buspirone. (**G**) and (**J**) were created in the free Plotly Make Chart program (https://chart-studio.plotly.com/create/#/). (**I**) was created by using LEfSe analysis tool from the galaxy site (https://huttenhower.sph.harvard.edu/galaxy/). Data values were indicated as mean ± SD (n = 6). Same letters are not significantly different (*p* < 0.05).

Exposure to EC significantly induced colitis in mice: it induced colon shortening, myeloperoxidase activity, and TNF-α, IL-1β, and IL-6 expression in the colon (Fig. 4A–E). However, treatments with buspirone suppressed EC*-*induced colon shortening, myeloperoxidase activity, and NF-κB activation in the colon, resulting in the attenuation of colitis.

Exposure to EC also raised gut microbiota alteration in mice: it shifted the β-diversity, while the α-diversity was not affected (Fig. 4F,G). Furthermore, it increased Proteobacteria and Firmicutes populations and reduced Bacteroidetes population (Fig. 4H, Supplementary Fig. S4, S5). Buspirone treatments changed EC*-*shifted β-diversity in the gut microbiota, while the α-diversity was not affected (Fig. 4F,G). Buspirone treatments also reduced EC-induced Proteobacteria population and increased Firmicutes and Tenericutes populations (Fig. 4H). Oral gavage of buspirone increased EC-suppressed Ruminococaceae population at the family level, PAC001091_g population at the genus level, and PAC001558_s population at the species level and reduced EC-induced Enterobacteriaceae population at the family, PAC000186_g population at the genus level, and PAC001064_s and PAC001065_s group populations at the species level (Supplementary Tables S4–6, Fig. 4, Supplementary Fig. S4). Interaperitoneal injection of buspirone increased EC-suppressed Bactreoidaceae population at the family level, Bacteroides population at the genus level, and PAC002443_s population at the species level and reduced EC-induced Helicobacteriaceae and Rikenellaceae populations at the family, Alistipes, PAC002367_g population at the genus level, and EU791023_s and PAC002367_s populations at the species level (Supplementary Tables S4–6, Fig. 4, Supplementary Fig. S6).

Next, we visualized the gut microbiota composition of mice treated with and without buspirone as a heat map at the genus level (Fig. 4J). Oral gavage or intraperitoneal injection of buspirone reduced EC-induced Escherichia population. Intraperitoneal injection of buspirone increased EC-induced Bacteroides population and decreased EC-induced PAC001228_g and PAC002367_g populations. Oral gavage of buspirone increased Odoribacter population. To understand what kinds of gut microbiota are associated with the therapeutic effects of buspirone in mice with IS-induced anxiety/depression, we analyzed the correlation between anxiety-/depression-like behaviors and gut microbiota in mice treated with and without buspirone (Fig. 4K). Lachnospiraceae (R = − 0.468, R^2^ = 0.219, p = 0.021), KE159600_g (R = − 0.613, R^2^ = 0.375, p = 0.002), LLKB_g (R = − 0.491, R^2^ = 0.241, p = 0.015), Helicobacter (R = − 0.676, R^2^ = 0.457, p = 0.005) and PAC001228_g populations (R = − 0.478, R^2^ = 0.228, p = 0.018) showed the negative correlation with the anxiety-like behavior (TL) in the LDT task, while PAC001066_g population (R = 0.464, R^2^ = 0.215, p = 0.022) showed the positive correlation. Bacteroides population (R = 0.549, R^2^ = 0.302) had the positive correlation with the anxiety-like behavior (OT) in the EPM. Roseburia population (R = 0.476, R^2^ = 0.227, p = 0.019) had the positive correlation with the depression-like behavior in TST.

### Effects of fecal microbiota transplantation (FMT) of buspirone-treated and untreated mice on IS-induced anxiety/depression in mice

**Figure 5:**
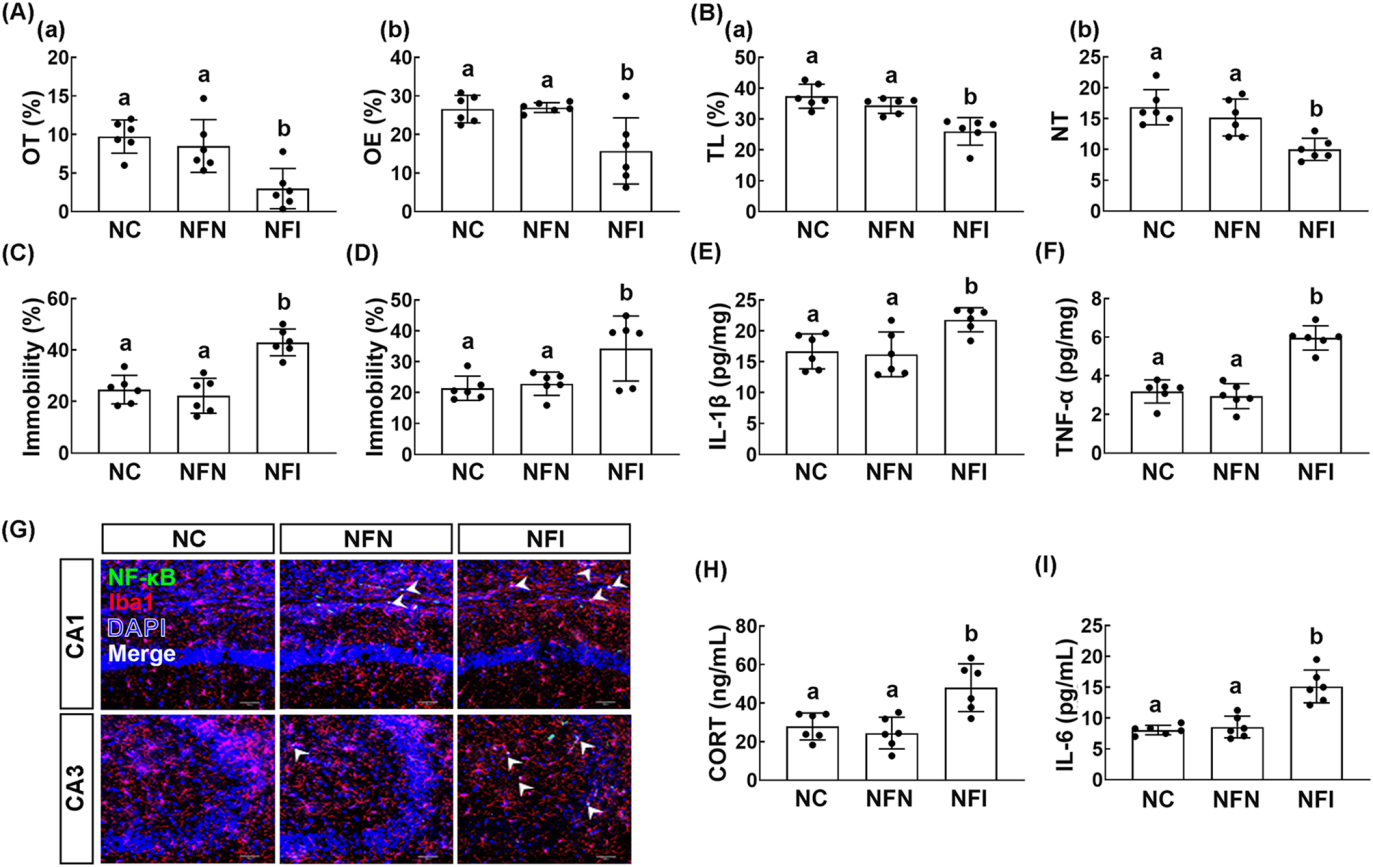
Effects of fecal transplantations of immobilization stress (IS)-treated and untreated control mice on the occurrence of anxiety/depression in the transplanted mice. Effects on IS-induced anxiety/depression-like behaviors in EPM task ((**A**) a: time spent in open arm [OT]; b: open arm entries [OE]), LDT ((**B**) a: time spent in the light box [TL]; b: number of transition into the light box entry [NT]), TST (**C**), and FST (**D**). Effects on IL-1β (**E**) and TNF-α expression (**F**) and NF-κB^+^/Iba1^+^ cell population (**G**) in the hippocampus. Effect on corticosterone (**H**) and IL-6 (**I**) in the blood. NFN and NFI groups were treated with the feces of normal mice (FN) and IS-treated mice (FI) daily for 5 days, respectively. NC group was orally treated with vehicle (saline) instead of the fecal sample. Data values were indicated as mean ± SD (n = 6). Same letters are not significantly different (*p* < 0.05).

In order to examine whether the alteration of gut microbiota by exposure to IS could cause anxiety/depression, we orally transplanted the feces of mice exposed to IS (FI) or normal control mice feces transplanted (FN) in normal mice and measured the anxiety-/depression-like behaviors in the transplanted mice (Fig. 5). FI transplantation significantly reduced the OT in the EPM task and TL in the LDT task and increased the immobility time in the TST and FST. FI transplantation increased TNF-α, IL-1β, and IL-6 expression and NF-κB^+^/Iba1^+^ cell population in the hippocampus. FI transplantation also increased corticosterone and IL-6 levels in the blood. Furthermore, FI transplantation induced colon shortening, increased myeloperoxidase activity, IL-1β, TNF-α and IL-6 expression, and NF-κB^+^/CD11c^+^ cell population in the colon (Supplementary Fig. 6). However, the FN transplantation did not affect the occurrence of anxiety/depression and colitis in the transplanted mice.

**Figure 6:**
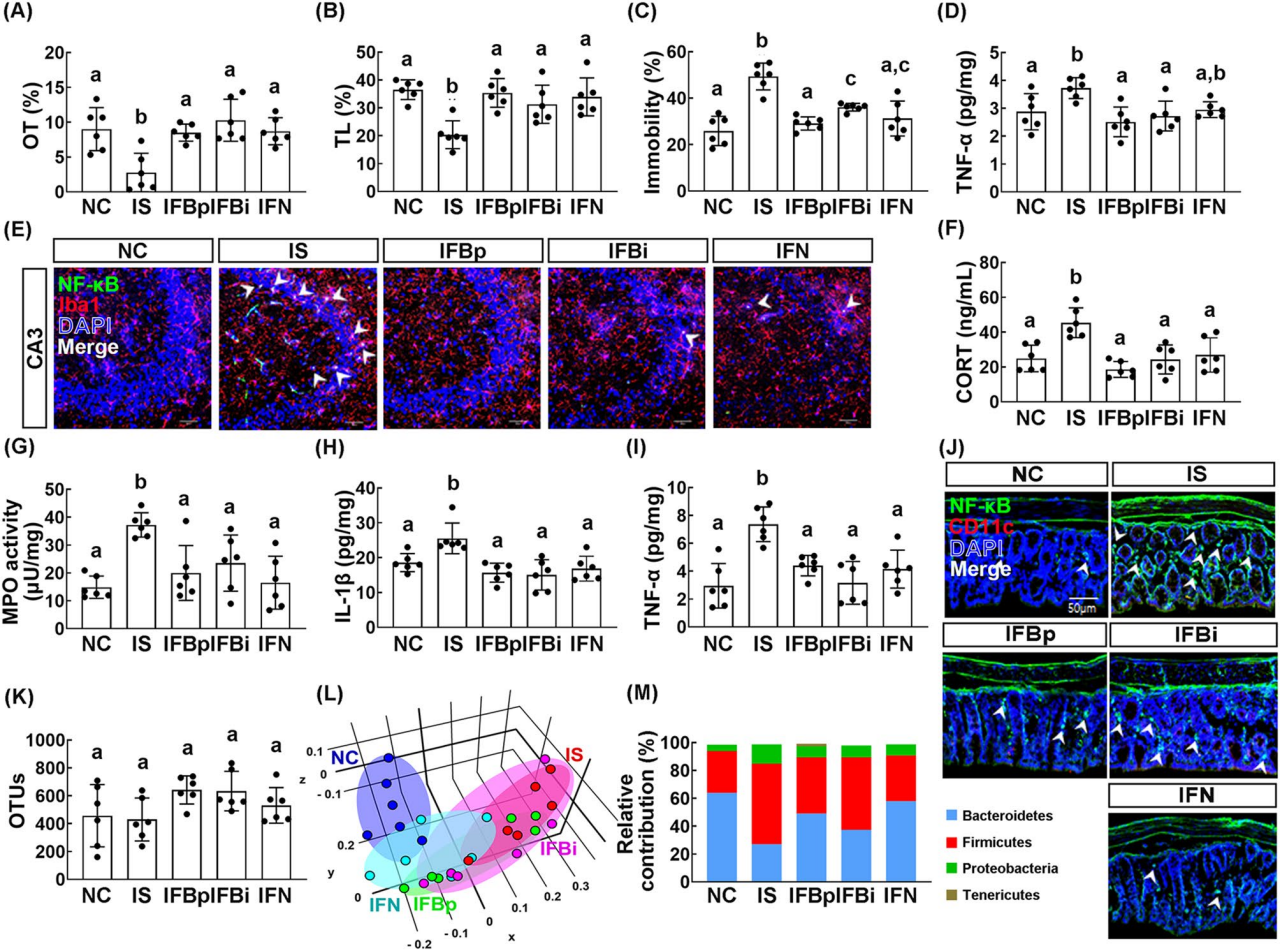
Fecal transplantation of buspirone/immobilization stress (IS)-treated mouse feces or untreated control mouse feces alleviated immobilization stress-induced anxiety/depression, colitis, and gut microbiota pertubation in the transplanted mice. Effects on IS-induced anxiety/depression-like behaviors in EPM task (**A**, OT), LDT (**B**, TL), and TST (**C**). Effects on TNF-α expression (**D**) and NF-κB^+^/Iba1^+^ cell population (**E**) in the hippocampus. Effect on corticosterone (CORT) level (**F**) in the blood. Effects on IS-induced myeloperoxidase (MPO) activity (**G**), IL-1β (**H**) and TNF-α expression (**I**), and NF-κB^+^/CD11c^+^ cell population (**J**) in the colon. Effects on the composition of gut microbiota: OTUs (**K**), principal coordinate analysis (PCoA) plot (**L**), and phylum (**M**). IFN, IFBp, and IFBi groups were orally gavaged with the feces of normal mice (FN), orally buspirone-gavaged mice with IS (FBp), and intraperitoneally buspirone-injected mice with IS (FBi) daily for 5 days from the next day after the final exposure to IS. A IS group was treated with vehicle (saline) in IS-exposed mice instead of fecal suspensions. Normal control mice (NC) was orally treated with vehicle (saline) instead of the fecal suspension. The present fecal microbiota compositions of NC and IS groups were shared in Fig. 2. (**L**) was created in the free Plotly Make Chart program (https://chart-studio.plotly.com/create/#/). Data values were indicated as mean ± SD (n = 6). Same alphabets are not significantly different (*p* < 0.05).

Next, to understand the role of gut microbiota on the anti-depressant activity of buspirone, we orally transplanted the feces of orally buspirone-treated IS mice (FBp), intraperitoneally buspirone-treated IS mice (FBi), or normal control mice (FN) into mice with IS. The transplantation of FBp, FBi, or FN suppressed IS-induced anxiety-/depression-like behaviors in the transplanted mice: they increased IS-suppressed OT in the EPM task and TL in the LDT task and suppressed the immobility time in the TST and FST (Fig. 6A–C, Supplementary Fig. S7A–C). They also suppressed IS-induced TNF-α and IL-1β expression and NF-κB^+^/Iba1^+^ cell population in the hippocampus (Fig. 6D,E, Supplementary Figs. S7D,E, S8A,B). The transplantations of FBp, FBi, and FN also suppressed IS-induced corticosterone and IL-6 levels in the blood (Fig. 6F, Supplementary Fig. S7F). They also alleviated IS-induced colitis: they suppressed colon shortening, myeloperoxidase activity, NF-κB^+^/CD11c^+^ cell population activation and IL-6 expression in the colon (Fig. 6G–I, Supplementary Fig. S7G,H).

FBp, FBi, or FN transplantation partially restored IS-shifted β-diversity in the gut microbiota to that of control mice, while the α-diversity was not significantly affected in the transplanted mice (Fig. 6K,L). They also suppressed IS-induced Proteobacteria and Firmicutes populations and increased Bacteroidetes population (Fig. 5M, Supplementary Figs. S7I, S9). In particular, FBp transplantation reduced IS-induced Christensenellaceae, Helicobacteriaceae, and Lachnospiraceae populations at the family level, Helicobacter and PAC000664_g, PAC001228_g populations at the genus level, and *Helicobacter rodentium* group at the species level and increased IS-suppressed Bacteroidaceae population at the family, Alistipes and PAC000664_g population at the genus level, and PAC000198_s and PAC002445_s populations at the species level (Supplementary Tables S7–S9, Supplementary Figs. S8, S9). FBi transplantation reduced IS-induced Helicobacteriaceae and Ruminococeae populations at the family level, Helicobacter, Oscillibacter and PAC000664_g populations at the genus level, *Helicobacter rodentium* group and PAC002027_s populations at the species level and increased IS-suppressed Muribaculaceae population at the family, Alistipes and PAC001068_g PAC000664_g populations at the genus level, and AB599946_s and PAC002445_s at the species level. FN transplantation reduced IS-induced Helicobacteriaceae, Lachnospiraceae, and Ruminococaceae populations at the family level, LLKB_g, Oscillibacter, PAC000664_g and Pseudoflavonifractor populations at the genus level, and Helicobacter rodentium group and PAC001083_s population at the species level and increased IS-suppressed Bacteroidaceae Muribaculaceae populations at the family, Alistipes population at the genus level, and EF097112_s and PAC002445_s at the species level.

## Discussion

Exposure to stressors including immobilization, antibiotics, or pathogen infection causes anxiety-like behaviors in rodents and induced the expression of proinflammatory cytokines TNF-α, IL-1β, and IL-6 in the hippocampus and colon, resulting in the occurrence of anxiety/depression and colitis^4,18,19^. In the present study, we also found that exposure to stressors IS and EC caused anxiety-/depression-like behaviors. Furthermore, they induced TNF-α, IL-1β, and IL-6 expression NF-κB^+^/Iba1^+^ cell population in the hippocampus and corticosterone and IL-6 levels in the blood, resulting in anxiety/depression through the regulation of neuroinflammation. Their exposures also increased TNF-α and IL-6 expression and NF-κB^+^/Iba1^+^ cell population in the colon and Proteobacteria population in the gut microbiota, leading to colitis and gut microbiota perturbation. Of Proteobacteria, many bacteria such as *Escherichia coli, Klebsiella pneumoniae*, *Klebsiella oxyto*ca, and *Proteus mirabilis* cause colitis and/or anxiety^10,20,21^. We also found that transplantation of IS-exposed mouse feces (FI), which exhibited a higher abundance of Proteobacteria population than normal control mice, caused anxiety-/depression-like behaviors, neuroinflammation, and colitis in the transplanted mice. These findings suggest that exposure to stressors including IS and EC can cause neuroinflammation, which can cause gut microbiota perturbation with colitis, and stressor-induced gut microbiota perturbation can deteriorate stressor-induced anxiety/depression.

In the present study, buspirone, which was orally gavaged or intraperitoneally injected, alleviated IS- or EC-induced anxiety-/depression-like behaviors. It also reduced TNF-α, IL-1β, and IL-6 expression and NF-κB-activated immune cell populations in the hippocampus and corticosterone and IL-6 levels in the blood. Furthermore, orally gavaged or intraperitoneally injected buspirone alleviated colitis: it reduced myeloperoxidase activity, TNF-α and IL-6 expression, and NF-κB^+^/CD11c^+^ cell population. In addition, Jang et al. reported that the induction of gut inflammation by exposure to IS or ampicillin caused anxiety/depression in mice^9,10^. The prevalence of anxiety/depression is significantly higher in patients with inflammatory bowel disease (IBD) than in healthy people^22,23^. Anti-IBD and anti-psychiatric therapies can alleviate psychiatric disorders and IBD, respectively^9,24,25^. *Lactobacillus rhamnosus* and *Lactobacillus johnsonii*, commensal gut lactobacilli, alleviate psychiatric disorder and colitis in mice^9,26^. These results suggest that buspirone can alleviate anxiety and depression, dependently on the suppression of colitis.

We also found that exposure to IS or EC altered gut microbiota composition in mice: they increased Proteobacteria population and decreased Bacteroidetes population in the feces. However, orally gavaged or intraperitoneally injected buspirone partially reversed IS- or EC-altered gut microbiota composition to that of normal control mice: it suppressed Proteobacteria populations and increased Bacteroidetes populations. Recent studies have focused that gut microbiota play pivotal roles in the neurodevelopmental processes and brain functions through the regulation of the MGB^27,28^. Dysregulation of the MGB by endogenous and exogenous stressors such as social defeat, aging, and pathogen infection accelerates the occurrence of gut inflammation with psychiatric disorders such as depression, anxiety, and Alzheimer’s disease^29,30^. These results suggest that buspirone can alleviate anxiety/depression and colitis through the modulation of gut microbiota composition. Therefore, to understand gut microbiota involved in the therapeutic effects of buspirone against anxiety/depression, we analyzed the correlation between anxiety-/depression-like behaviors and gut microbiota in mice treated with and without buspirone. The anxiolytic activity of buspirone was positively correlated with the populations of Bacteroides (R = 0.433, R^2^ = 0.187, p = 0.002) and PAC001066_g (R = 0.550, R^2^ = 0.302, p =  < 0.001) in EC- or IS-exposed mice, while the populations of Lachnospiraceae (R = − 0.412, R^2^ = 0.170, p = 0.004), KE159660_g (R = − 0.444, R^2^ = 0.197, p = 0.002), LLKB_g (R = − 0.454, R^2^ = 0.206, p = 0.001), Helicobacter (R = − 0.311, R^2^, 0.097, p = 0.031), and PAC001228_g (R = − 0.467, R^2^ = 0.218, p = 0.001) was negatively correlated (Supplementary Fig. S5). The anti-depressive activity of buspirone was positively correlated with the Roseburia population (R = 0.373, R^2^ = 0.139, p = 0.009) (Supplementary Fig. S6). These findings support the suggestion that the gut microbiota composition is pivotal in the attenuation of anxiety/depression.

Many studies have reported that buspirone can improve anxiety and depression in mice and humans by activating 5-HT1A autoreceptors and 5-HT1A heteroreceptors, respectively^13,15,16,31–34^. Many drugs modulate gut microbiota composition^35,36^. Therefore, in the present study, the anxiolytic, anti-depressant, and anti-neuroinflammatory effects of buspirone may be due to the activation of 5-HT1A receptors in the brain and spinal cord, resulting in the improvement of gut microbiota composition. Therefore, to clarify the role of gut microbiota in the attenuation of anxiety/depression and neuroinflammation by buspirone, we transplanted the gut microbiota of buspirone-treated mice with IS or normal control mice in mice with IS. The transplantation of FBp, FBi, or FN mitigated IS-induced colitis in mice: they alleviated IS-induced myeloperoxidase and TNF-α and IL-6 expression in the colon. These FMTs also partially reversed IS-altered gut microbiota composition to that of normal control mice: they suppressed Proteobacteria and Firmicutes populations and increased Bacteroidetes population. Furthermore, fecal microbiota transplantations suppressed IS-induced TNF-α, IL-1β, and IL-6 expression and NF-κB^+/^Iba1^+^ cell population in the hippocampus, resulting in the amelioration of anxiety/depression. In addition, exposure to stressors causes anxiety-like behaviors in germ-free mice more exaggeratedly than in specific pathogen-free (SPF) ones^37^. However, the FMT from SPF mice into germ-free mice alleviates the hyper-responsive anxiety-like behaviors and reduces blood corticosterone^38^. These findings suggest that buspirone can modulate anxiety/depression and colitis by modulating the composition of gut microbiota and activation of 5-HT1A receptors.

In conclusion, the induction of gut microbiota perturbation by exposure to stressors including IS and EC can cause anxiety and depression and buspirone alleviates IS as well as EC-induced anxiety/depression and colitis. It also suppresses associated neuroinflammation and modulates gut microbiota. Future studies can help to explain the relationship, if any, in the central and peripheral effects of buspirone.

## Materials and methods

### Materials

Buspirone and tetramethylbenzidine were purchased from Sigma-Aldrich (St Louis, MO).

An antibody for Iba1, Alexa Fluor 488, and Alexa Fluor 594 were purchased from Invitrogen (Carlsbad, CA). An antibody for NF-κB were purchased from Cell Signaling Technology (Beverly, MA). Enzyme-linked immunosorbent assay (ELISA) kits for TNF-α, IL-1, and IL-6 were purchased from Ebioscience (Atlanta, GA). An ELISA kit for corticosterone was purchased from Elabscience (Hebei, China). A QIAamp DNA stool mini kit was purchased from Qiagen (Hilden, Germany).

### Animals

C57BL/6N mice (male, 19 ~ 21 g, 6 weeks old) were supplied from Samtaco Inc. (Gyunggi-do, Korea) and acclimated for 1 week before experiments. Mice (three mice per cage) were maintained in wire cages (length 50 × width 30 × height 20 cm) with the 5-cm raised wire floor, which was designed to prohibit mice for feeding the feces, under a ventilated condition (temperature, 20–22 °C; humidity, 50% ± 10% humidity; light/dark cycle, 12-h/12-h; and cleaning, 24 h) and fed standard laboratory chow and water ad libitum.

### Generation of mice with anxiety/depression

Mice with anxiety/depression were prepared by exposure to IS or EC, as previously reported^20,39^. All animal experiments were conducted with two replicates. Each group contained six mice.

First, to understand the effect of buspirone on anxiety/depression, buspirone (dissolved in saline) was orally gavaged (5 mg/kg/day) or intraperitoneally injected (1 mg/kg/day) in mice with IS- or EC-induced anxiety/depression once a day for 5 days. Other groups (NC, normal control mice; IS, IS-treated mice; and EC, EC-treated mice) were treated with vehicle (saline) instead of buspirone.

Second, to understand the effects of gut microbiota on the occurrence of anxiety/depression, fecal suspensions (NFN, vehicle-treated mouse fecal suspension; and NFI, IS-induced mouse fecal suspension) were transplanted into mice with IS-induced depression. Other groups (NC, normal control mice; and IS, IS-treated mice) were treated with vehicle (saline) instead of fecal suspension.

Third, to understand the gut microbiota-mediated effect of buspirone on the anxiety/depression, fecal suspensions (IFN, vehicle-treated mouse fecal suspension; IFBp, orally buspirone-treated mouse fecal suspension; and IFBpi, intraperitoneally buspirone-treated mouse fecal suspension) were transplanted into mice with IS-induced depression. Normal control mice (NC) and IS-treated mice (IS) were treated with vehicle (saline) instead of fecal transplantation.

Anxiety/depression-related behaviors were measured in the EPM, LDT tasks, TST, and FST. At the end of the behavioral test, all animals were sacrificed. Bloods, brains, and colons were collected. Brain and colon were stored at − 80 °C for the assay of biochemical markers. For the immunohistochemistry assay, mice were trans-cardiacally perfused with 4% paraformaldehyde for brain and colon tissue fixation. Brains and colon tissues were post-fixed with 4% paraformaldehyde for 4 h, cytoprotected in 30% sucrose solution, freezed, and cut using a cryostat^20,39^.

### Preparation of the fecal suspension for FMT

The fresh FBp and FBi (0.5 g) for FMT were collected 48 h after the final *p.o.* or *i.p*. treatment with buspirone in mice with IS-induced anxiety/depression to gather the feces not containing orally administered buspirone, respectively. The fresh FN (0.5 g) was collected 48 h after the final treatment with vehicle in NC. The FI (0.5 g) was collected 48 h after the final treatment with vehicle in mice with IS-induced anxiety/depression. The collected feces were suspended in saline on ice and centrifuged at 500*g* for 5 min at 4 °C. The resulting supernatant was centrifuged at 10,000*g* for 15 min at 4 °C, washed with saline twice, and suspended in saline (4.5 mL). The suspensions were used for further fecal transplantation.

### Behavioral task

The EPM task was performed in the plus-maze apparatus (consisting of two open [30 × 7 cm] and two enclosed arms [30 × 7 cm] with 20-cm-high walls extending from a central platform [7 × 7 cm] on a single central support to a height of 60 cm above the floor) for 5 min, as previously reported^20,39^. The LDT task was assessed in the light (350 lx)/dark box (2 lx) apparatus (45 × 25 × 25 cm) for 8 min according to the method of Jang et al.^9^. This maze had two chambers with black and white polywood walls and Plexiglass for floors. These chambers were connected by an opening (7.5 × 7.5 cm) located in the center of the dividing wall at floor level. The TST was measured according to the method of Kim et al.^39^. Mice were suspended on the edge of a table 30 cm above the floor by taping 1 cm from the tail tip. Immobility time was measured for 5 min. Mice were judged to be immobile, when they did not move and hanged passively. The FST was performed in a round transparent plastic jar (20 × 40 cm^3^) containing fresh water (25 °C) to a height of 25 cm according to the method of Kim et al.^39^. Immobility time was measured during 5 min. Mice were judged to be immobile, when they remained floating in the water without struggling.

### Myeloperoxidase activity assay and ELISA

Myeloperoxidase activity was assayed according to the method of Jang et al.^9^. Colon tissues were homogenized with cold radioimmunoprecipitation assay lysis buffer and centrifuged at 10,000*g* for 10 min. The supernatant was used as a crude enzyme solution. An aliquot (0.05 mL) of the supernatant was added in the reaction mixture (0.95 mL) containing 0.03% hydrogen peroxide and 1.6 mM tetramethylbenzidine. The absorbance at 650 nm was monitored over 5 min. Activity was defined as the quantity degrading 1 μmol/mL of peroxide.

For the cytokine assay, the supernatants of the hippocampus and colon homogenate were transferred to 96-well plates according to the method of Kim et al.^39^. TNF-α, IL-1β, and IL-6 concentrations were determined in the supernatant using commercial ELISA kits. For the corticosterone assay, the plasma was prepared according to the method of Kim et al.^39^. Corticosterone concentration was measured in the plasma using an ELISA kit^9^.

### Immunofluorescence assay

The hippocampus and colon tissues of mice were trans-cardiacally perfused with 4% paraformaldehyde, post-fixed with 4% paraformaldehyde, cytoprotected in 30% sucrose solution, freezed, and cryosectioned, as previously reported^39,40^. The hippocampus tissue sections were incubated with the NF-κB and Iba1 antibodies for activated microglia and colon tissue sections were incubated with NF-κB and CD11c antibodies for NF-κB^+^/CD11c^+^ cells (activated dendritic cells and macrophages). These sections were then treated with secondary antibodies conjugated with Alexa Fluor 488 (1:1000) or Alexa Fluor 594 (1:500). The stained slices were observed with a confocal laser microscope.

### Bacterial 16S rRNA gene sequencing

The fresh stools of mice were collected and their bacterial genomic DNAs were extracted using a QIAamp DNA stool mini kit, as previously reported^39^. Amplification of genomic DNA was performed using barcoded primers targeted the bacterial 16S rRNA V4 region gene. Each amplicon was sequenced using Illumina iSeq 100 (San Diego, CA). Prediction for functional genes was analyzed using the phylogenetic investigation of communities by reconstruction of unobserved states^31,39,41^. The pyrosequencing reads were deposited in the short read archive of NCBI under accession number PRJNA507690.

### Statistical analysis

All data are expressed as the means ± standard deviation (SD) and conducted GraphPad Prism 8 (GraphPad Software, Inc., San Diego, CA, USA). The significance was analyzed by Tukey's multiple comparisons and Dunnett’s multiple comparisons, using one-way ANOVA in SPSS (*p* < 0.05). All p-values in the present study are indicated in Supplementary Table S10.

### Ethics statement

All animal experiments were approved by the Institutional Animal Care and Use Committee of the University (IACUC No KUASP(SE)-19-152) and were performed according to the NIH, AAALAC International, and University Guide for Laboratory Animals Care and Usage.

## Supplementary Information

Supplementary Information.
