# Supplementary material for: Buspirone alleviates anxiety, depression, and colitis; and modulates gut microbiota in mice
Source: Sci Rep. 2021 Mar 17;11:6094. doi: 10.1038/s41598-021-85681-w (PMC7969772; doi:10.1038/s41598-021-85681-w)
Supplement: Supplementary file 1 — Supplementary Information. [file 41598_2021_85681_MOESM1_ESM.docx]

**[Supplement]**

**Buspirone alleviates anxiety, depression, and colitis; and modulates gut microbiota in mice**

Jeon-Kyung Kim^#^, Sang-Kap Han^#^, Min-Kyung Joo, and Dong-Hyun Kim^*^

*Neurobiota Research Center, College of Pharmacy, Kyung Hee University, 26, Kyungheedae-ro, Dongdaemun-gu, Seoul 02447, Korea*

Table S1. Effects of orally gavaged (ISBp) or intraperitoneally injected buspirone (ISBi) on the gut microbiota composition at the family level in mice with immobilization stress (IS)

| Taxon Name | Relative contribution (%) | | | |
| --- | --- | --- | --- | --- |
|  | NC | IS | ISBp | ISBi |
| Akkermansiaceae | 0.73 ± 1.11 | 0.01 ± 0.02 | 0.01 ± 0.01 | 1.67 ± 2.44 |
| Bacteroidaceae | 12.76 ± 7.74 | 3.08 ± 2.35^#^ | 5.97 ± 4.21 | 4.86 ± 3.41 |
| Desulfovibrionaceae | 1.35 ± 0.83 | 2.35 ± 2.48 | 0.95 ± 0.43 | 1.23 ± 1.27 |
| Helicobacteraceae | 2.79 ± 1.30 | 11.29 ± 7.38^#^ | 3.62 ± 1.49* | 9.72 ± 11.15 |
| Lachnospiraceae | 19.67 ± 3.56 | 40.54 ± 11.08^#^ | 27.58 ± 10.78 | 26.57 ± 13.66 |
| Muribaculaceae | 25.75 ± 10.10 | 15.32 ± 9.46 | 36.14 ± 5.46* | 26.27 ± 18.74 |
| Mycoplasmataceae | 0.00 ± 0.00 | 0.66 ± 0.98 | 0.24 ± 0.39 | 1.27 ± 2.67 |
| Prevotellaceae | 17.99 ± 8.42 | 3.40 ± 2.58^#^ | 5.81 ± 3.56 | 4.22 ± 3.19 |
| Rikenellaceae | 5.62 ± 0.43 | 3.55 ± 1.57^#^ | 6.81 ± 4.24 | 6.95 ± 5.37 |
| Ruminococcaceae | 9.33 ± 2.96 | 15.40 ± 2.67^#^ | 8.10 ± 1.46* | 11.42 ± 4.53 |

Values indicate means±SD.  ^#^*p*<0.05 vs. NC group. **p*<0.05 vs. IS group.

Table S2. Effects of orally gavaged (ISBp) or intraperitoneally injected buspirone (ISBi) on the gut microbiota composition at the genus level in mice with immobilization stress

| Taxon Name | Relative contribution (%) | | | |
| --- | --- | --- | --- | --- |
|  | NC | IS | ISBp | ISBi |
| Akkermansia | 0.73 ± 1.11 | 0.01 ± 0.02 | 0.01 ± 0.01 | 1.67 ± 2.44 |
| Alistipes | 5.40 ± 0.49 | 3.23 ± 1.39^#^ | 6.30 ± 4.01 | 6.61 ± 5.30 |
| Bacteroides | 12.75 ± 7.71 | 3.08 ± 2.35^#^ | 5.96 ± 4.20 | 4.86 ± 3.41 |
| Eubacterium_g23 | 1.21 ± 1.03 | 0.17 ± 0.08^#^ | 0.16 ± 0.10 | 0.36 ± 0.55 |
| Helicobacter | 2.79 ± 1.30 | 11.28 ± 7.36^#^ | 3.61 ± 1.48* | 9.63 ± 10.99 |
| KE159538_g | 4.54 ± 4.52 | 7.33 ± 6.23 | 13.78 ± 9.30 | 4.68 ± 5.47 |
| LLKB_g | 0.62 ± 0.61 | 2.22 ± 0.75^#^ | 0.77 ± 0.57* | 1.93 ± 1.26 |
| Muribaculum | 1.75 ± 0.99 | 0.65 ± 0.47^#^ | 1.71 ± 1.00* | 1.59 ± 1.39 |
| Odoribacter | 0.34 ± 0.49 | 0.98 ± 1.04 | 1.14 ± 0.33 | 1.15 ± 2.11 |
| Oscillibacter | 1.61 ± 0.75 | 4.88 ± 0.91^#^ | 2.81 ± 0.78* | 2.93 ± 2.11 |
| PAC000186_g | 6.47 ± 3.21 | 5.10 ± 3.50 | 10.16 ± 2.59* | 7.22 ± 5.77 |
| PAC000198_g | 2.22 ± 2.37 | 1.03 ± 0.53 | 2.08 ± 0.37* | 1.64 ± 1.35 |
| PAC000664_g | 2.98 ± 3.42 | 6.07 ± 3.98 | 1.21 ± 0.46* | 2.01 ± 1.56* |
| PAC001068_g | 7.45 ± 3.14 | 3.97 ± 2.30 | 9.21 ± 4.00* | 8.84 ± 8.46 |
| PAC001091_g | 2.94 ± 2.83 | 4.10 ± 4.09 | 1.82 ± 2.03 | 2.07 ± 2.00 |
| PAC001228_g | 0.97 ± 0.88 | 3.23 ± 1.86^#^ | 1.38 ± 0.86 | 0.88 ± 1.08* |
| PAC001765_g | 0.72 ± 0.83 | 0.56 ± 0.55 | 2.50 ±1.38* | 0.78 ± 0.60 |
| PAC002367_g | 1.23 ± 2.16 | 5.01 ± 8.88 | 0.57 ± 0.70 | 1.15 ± 2.13 |
| Paraprevotella | 3.53 ± 3.31 | 0.80 ± 0.90 | 1.40 ± 1.79 | 2.20 ± 2.72 |
| Prevotella | 10.08 ± 10.59 | 1.12 ± 1.01 | 2.56 ± 1.74 | 0.90 ± 0.77 |
| Prevotellaceae_uc | 3.67 ± 3.67 | 1.23 ± 2.15 | 0.32 ± 0.17 | 0.44 ± 0.68 |
| Pseudoflavonifractor | 2.75 ± 0.97 | 5.81 ± 2.63^#^ | 2.56 ± 0.30* | 3.70 ± 2.10 |
| Ruminococcus | 1.46 ± 1.21 | 0.64 ± 1.07 | 0.42 ± 0.39 | 1.11 ± 1.18 |

Values indicate means±SD.  ^#^*p*<0.05 vs. NC group. **p*<0.05 vs. IS group.

Table S3. Effects of orally gavaged (ISBp) or intraperitoneally injected buspirone (ISBi) on the gut microbiota composition at the species level in mice with immobilization stress

| Taxon Name | Relative contribution (%) | | | |
| --- | --- | --- | --- | --- |
|  | NC | IS | ISBp | ISBi |
| AB599946_s | 5.34 ± 3.04 | 1.16 ± 1.23^#^ | 2.79 ± 2.50 | 3.33 ± 2.85 |
| Bacteroides acidifaciens group | 3.78 ± 2.65 | 1.23 ± 0.97 | 2.21 ± 1.50 | 0.85 ± 0.44 |
| EF097112_s | 2.84 ± 1.74 | 1.44 ± 1.18 | 2.56 ± 1.12 | 2.12 ± 1.37 |
| EU622763_s group | 7.63 ± 11.22 | 0.28 ± 0.47 | 0.22 ± 0.28 | 0.02 ± 0.02 |
| FJ880724_s | 3.37 ± 3.09 | 0.80 ± 0.90 | 1.39 ± 1.77 | 2.17 ± 2.69 |
| Helicobacter japonicus | 1.03 ± 0.57 | 2.53 ± 2.09 | 1.51 ± 0.96 | 1.79 ± 1.97 |
| Helicobacter rodentium group | 1.74 ± 0.83 | 8.70 ± 7.51^#^ | 2.08 ± 1.47 | 7.68 ± 11.20 |
| KE159538_g_uc | 1.97 ± 4.76 | 2.43 ± 4.95 | 3.84 ± 5.99 | 0.09 ± 0.09 |
| PAC001064_s | 2.08 ± 1.95 | 1.43 ± 1.34 | 3.78 ± 1.64* | 2.51 ± 3.21 |
| PAC001065_s group | 3.24 ± 1.66 | 3.26 ± 2.18 | 5.81 ± 1.42* | 3.95 ± 2.70 |
| PAC001070_s group | 1.31 ± 1.14 | 1.20 ± 1.34 | 3.30 ± 3.49 | 4.09 ± 5.55 |
| PAC001083_s | 0.56 ± 0.81 | 2.42 ± 2.63 | 0.18 ± 0.18 | 0.45 ± 0.70 |
| PAC001120_s | 0.03 ± 0.04 | 0.37 ± 0.76 | 6.46 ± 10.93 | 0.32 ± 0.69 |
| PAC001558_s | 2.40 ± 2.99 | 2.58 ± 4.29 | 1.72 ± 2.03 | 1.80 ± 1.56 |
| PAC002027_s | 0.67 ± 0.81 | 2.61 ± 2.33 | 0.57 ± 0.25 | 0.64 ± 1.12 |
| PAC002367_s | 1.23 ± 2.16 | 4.97 ± 8.80 | 0.57 ± 0.70 | 1.14 ± 2.10 |
| PAC002443_s | 1.97 ± 2.79 | 0.26 ± 0.28 | 0.48 ± 0.34 | 0.33 ± 0.42 |
| PAC002444_s | 0.99 ± 0.61 | 1.49 ± 0.98 | 3.41 ± 3.80 | 2.86 ± 2.74 |
| PAC002445_s | 2.83 ± 0.49 | 0.79 ± 0.50^#^ | 1.17 ± 0.98 | 1.30 ± 1.07 |
| PAC002478_s | 1.20 ± 0.74 | 2.13 ± 2.42 | 0.71 ± 0.41 | 1.00 ± 1.10 |

Values indicate means±SD.  ^#^*p*<0.05 vs. NC group. **p*<0.05 vs. IS group.

Table S4. Effects of orally gavaged (ECBp) or intraperitoneally injected buspirone (ECBi) on the gut microbiota composition at the family level in mice with *Escherichia coli* K1 (EC)-induced depression

| Taxon Name | Relative contribution (%) | | | |
| --- | --- | --- | --- | --- |
|  | NC | EC | ECBp | ECBi |
| Bacteroidaceae | 13.62 ± 8.30 | 4.80 ± 1.51^#^ | 2.99 ± 1.61 | 9.23 ± 5.99 |
| Enterobacteriaceae | 0.00 ± 0.00 | 2.08 ± 4.97 | 0.79 ± 1.41 | 0.02 ± 0.03 |
| Helicobacteraceae | 2.83 ± 2.46 | 8.72 ± 5.04^#^ | 7.91 ± 2.35 | 5.44 ± 4.08 |
| Lachnospiraceae | 19.51 ± 11.46 | 28.50 ± 14.12 | 41.25 ± 10.27 | 33.43 ± 7.80 |
| Muribaculaceae | 22.34 ± 9.60 | 25.60 ± 12.21 | 12.68 ± 8.19 | 23.65 ± 7.37 |
| Mycoplasmataceae | 0.02 ± 0.03 | 1.64 ± 3.47 | 3.87 ± 3.60 | 0.40 ± 0.14 |
| Odoribacteraceae | 0.12 ± 0.17 | 0.92 ± 0.60^#^ | 1.86 ± 0.85 | 0.62 ± 0.66 |
| Prevotellaceae | 20.05 ± 11.14 | 6.30 ± 6.65^#^ | 0.95 ± 0.69 | 6.15 ± 4.03 |
| Rikenellaceae | 4.24 ± 1.66 | 6.68 ± 2.79 | 6.64 ± 2.09 | 3.61 ± 1.37* |
| Ruminococcaceae | 12.02 ± 4.72 | 9.00 ± 2.25 | 13.91 ± 2.71* | 10.35 ± 3.92 |

Values indicate means±SD.  ^#^*p*<0.05 vs. NC group. **p*<0.05 vs. EC group.

Table S5. Effects of orally gavaged (ECBp) or intraperitoneally injected buspirone (ECBi) on the gut microbiota composition at the genus level in mice with *Escherichia coli* K1 (EC)-induced depression

| Taxon Name | Relative contribution (%) | | | |
| --- | --- | --- | --- | --- |
|  | NC | EC | ECBp | ECBi |
| Alistipes | 4.04 ± 1.64 | 6.16 ± 2.80 | 5.71 ± 2.21 | 3.33 ± 1.28* |
| Alloprevotella | 3.66 ± 4.08 | 0.55 ± 0.87 | 0.10 ± 0.18 | 0.01 ± 0.01 |
| Bacteroides | 13.61 ± 8.30 | 4.80 ± 1.52^#^ | 2.99 ± 1.60 | 9.22 ± 5.98 |
| Escherichia | 0.00 ± 0.00 | 2.06 ± 4.92 | 0.79 ± 1.40 | 0.02 ± 0.03 |
| Eubacterium_g23 | 1.78 ± 1.11 | 0.16 ± 0.08^#^ | 0.07 ± 0.06* | 0.10 ± 0.06 |
| Helicobacter | 2.83 ± 2.45 | 8.70 ± 5.03^#^ | 7.89 ± 2.33 | 5.41 ± 4.03 |
| KE159538_g | 2.30 ± 1.71 | 3.16 ± 3.79 | 3.95 ± 1.05 | 15.67 ± 9.39* |
| LLKB_g | 0.72 ± 0.47 | 2.75 ± 1.61^#^ | 2.21 ±1.38 | 1.62 ± 2.83 |
| Odoribacter | 0.11 ± 0.17 | 0.92 ± 0.59^#^ | 1.85 ± 0.83 | 0.62 ± 0.66 |
| Oscillibacter | 1.92 ± 1.03 | 2.27 ± 0.99 | 5.48 ± 1.69* | 2.31 ± 1.81 |
| PAC000186_g | 4.89 ± 2.41 | 8.49 ± 4.20 | 3.94 ± 2.95 | 6.49 ± 2.93 |
| PAC000198_g | 2.05 ± 1.99 | 1.79 ± 1.01 | 1.12 ± 0.77 | 1.79 ± 0.69 |
| PAC000664_g | 3.29 ± 3.02 | 1.78 ± 1.11 | 3.04 ± 1.26 | 2.17 ± 2.00 |
| PAC001068_g | 5.45 ± 2.10 | 5.08 ± 2.67 | 2.36 ± 1.51 | 5.66 ± 2.01 |
| PAC001091_g | 4.26 ± 7.73 | 1.82 ± 0.85 | 5.44 ± 5.94 | 1.63 ± 0.93 |
| PAC001228_g | 0.99 ± 0.56 | 2.04 ± 1.66 | 3.37 ± 1.53 | 1.13 ± 1.21 |
| PAC002367_g | 0.07 ± 0.13 | 3.44 ± 2.71^#^ | 4.80 ± 4.74 | 0.18 ± 0.27* |
| Paraprevotella | 4.04 ± 2.92 | 1.20 ± 0.87^#^ | 0.18 ± 0.25* | 1.49 ± 1.04 |
| Prevotella | 9.93 ± 9.60 | 1.34 ± 1.41 | 0.25 ± 0.18 | 2.09 ± 0.82 |
| Prevotellaceae_uc | 2.42 ± 2.32 | 3.21 ± 5.00 | 0.42 ± 0.64 | 2.57 ± 3.08 |
| Pseudoflavonifractor | 3.10 ± 1.97 | 3.09 ± 1.54 | 3.70 ± 0.64 | 3.98 ± 1.71 |
| Ruminococcus | 1.87 ± 1.87 | 0.59 ± 0.53 | 0.29 ± 0.18 | 0.95 ± 1.53 |

Values indicate means±SD.  ^#^*p*<0.05 vs. NC group. **p*<0.05 vs. EC group.

Table S6. Effects of orally gavaged (ECBp) or intraperitoneally injected buspirone (ECBi) on the gut microbiota composition at the family level in mice with *Escherichia coli* K1 (EC)-induced depression

| Taxon Name | Relative contribution (%) | | | |
| --- | --- | --- | --- | --- |
|  | NC | EC | ECBp | ECBi |
| AB599946_s | 8.04 ± 7.07 | 2.29 ± 1.09 | 1.35 ± 0.99 | 3.31 ± 1.30 |
| Bacteroides acidifaciens group | 3.32 ± 1.56 | 1.43 ± 0.72^#^ | 1.35 ± 0.95 | 1.82 ± 1.56 |
| DQ777952_s | 2.09 ± 1.19 | 0.23 ± 0.29^#^ | 0.02 ± 0.04 | 0.43 ± 0.60 |
| EF097112_s | 1.31 ± 1.12 | 2.00 ± 1.27 | 1.03 ± 0.78 | 1.62 ± 0.66 |
| EU622763_s group | 7.52 ± 9.88 | 0.87 ± 1.01 | 0.03 ± 0.05 | 0.91 ± 0.90 |
| EU791023_s | 0.21 ± 0.22 | 2.30 ± 2.69 | 0.27 ± 0.22 | 0.39 ± 0.29 |
| Escherichia coli group | 0.00 ± 0.00 | 1.99 ± 4.74 | 0.78 ± 1.38 | 0.02 ± 0.03 |
| FJ880724_s | 3.22 ± 1.88 | 1.20 ± 0.87^#^ | 0.18 ± 0.25* | 1.48 ± 1.04 |
| Helicobacter japonicus | 1.37 ± 1.11 | 3.95 ± 2.48^#^ | 3.13 ± 0.71 | 2.79 ± 2.28 |
| Helicobacter rodentium group | 1.45 ± 1.36 | 4.67 ± 4.14 | 4.69 ± 1.93 | 2.54 ± 1.94 |
| Lactobacillus murinus group | 0.09 ± 0.09 | 0.44 ± 0.42 | 1.59 ± 1.92 | 0.57 ± 0.98 |
| Mycoplasma sualvi | 0.02 ± 0.03 | 1.59 ± 3.37 | 3.67 ± 3.35 | 0.40 ± 0.14 |
| PAC001064_s | 1.29 ± 0.67 | 2.46 ± 1.53 | 0.81 ± 0.62* | 1.60 ± 1.56 |
| PAC001065_s group | 1.81 ± 1.08 | 5.48 ± 2.94^#^ | 2.30 ± 2.03 | 4.46 ± 1.78 |
| PAC001558_s | 3.76 ± 7.80 | 1.48 ± 0.98 | 4.52 ± 5.47 | 0.38 ± 0.37* |
| PAC002367_s | 0.07 ± 0.13 | 3.42 ± 2.69^#^ | 4.75 ± 4.66 | 0.18 ± 0.27* |
| PAC002443_s | 0.82 ± 0.50 | 0.26 ± 0.15^#^ | 0.11 ± 0.10 | 3.41 ± 2.94* |
| PAC002444_s | 0.48 ± 0.34 | 2.49 ± 1.55^#^ | 3.16 ± 2.19 | 1.07 ± 1.37 |
| PAC002445_s | 2.40 ± 1.24 | 1.93 ± 1.18 | 0.35 ± 0.23* | 1.23 ± 0.76 |
| PAC002479_s | 3.32 ± 3.71 | 0.54 ± 0.85 | 0.10 ± 0.17 | 0.01 ± 0.01 |

Values indicate means±SD.  ^#^*p*<0.05 vs. NC group. **p*<0.05 vs. EC group.

Table S7. Effects of fecal transplantation of normal mice (FN), orally buspirone-gavaged mice with IS (FBp), and intraperitoneally buspirone-injected mice with IS (FBi) on the gut microbiota composition at the family level in mice with *Escherichia coli* K1 (EC)-induced depression

| Taxon Name | Relative contribution (%) | | | | |
| --- | --- | --- | --- | --- | --- |
|  | NC | IS | IFBp | IFBi | IFN |
| Bacteroidaceae | 12.76 ± 7.74 | 3.08 ± 2.35^#^ | 4.57 ± 2.45 | 4.56 ± 1.39 | 2.75 ± 2.10 |
| Christensenellaceae | 0.25 ± 0.19 | 1.11 ± 0.68^#^ | 0.57 ± 0.36 | 0.58 ± 0.36 | 1.44 ± 2.06 |
| Desulfovibrionaceae | 1.35 ± 0.83 | 2.35 ± 2.48 | 0.94 ± 1.10 | 0.93 ± 0.26 | 1.35 ± 0.78 |
| Helicobacteraceae | 2.79 ± 1.30 | 11.29 ± 7.38^#^ | 6.69 ± 6.21 | 7.28 ± 7.35 | 6.36 ± 4.90 |
| Lachnospiraceae | 19.67 ± 3.56 | 40.54 ± 11.08^#^ | 28.11 ± 17.14 | 40.82 ± 17.60 | 20.87 ± 7.53* |
| Muribaculaceae | 25.75 ± 10.10 | 15.32 ± 9.46 | 26.75 ± 12.44 | 19.88 ± 12.20 | 39.46 ± 14.11* |
| Odoribacteraceae | 0.34 ± 0.49 | 0.98 ± 1.04 | 1.42 ± 1.09 | 1.05 ± 0.35 | 0.98 ± 1.95 |
| Prevotellaceae | 17.99 ± 8.42 | 3.40 ± 2.58^#^ | 7.99 ± 6.20 | 5.79 ± 6.11 | 7.05 ± 4.74 |
| Rikenellaceae | 5.62 ± 0.43 | 3.55 ± 1.57^#^ | 6.57 ± 2.98 | 5.35 ± 3.37 | 6.16 ± 4.81 |
| Ruminococcaceae | 9.33 ± 2.96 | 15.40 ± 2.67^#^ | 10.48 ± 5.05 | 9.80 ± 2.23* | 9.42 ± 2.99* |

Values indicate means±SD.  ^#^*p*<0.05 vs. NC group. **p*<0.05 vs. IS group.

Table S8. Effects of fecal transplantation of normal mice (FN), orally buspirone-gavaged mice with IS (FBp), and intraperitoneally buspirone-injected mice with IS (FBi) on the gut microbiota composition at the genus level in mice with *Escherichia coli* K1 (EC)-induced depression

| Taxon Name | Relative contribution (%) | | | | |
| --- | --- | --- | --- | --- | --- |
|  | NC | IS | IFBp | IFBi | IFN |
| Alistipes | 5.40 ± 0.49 | 3.23 ± 1.39^#^ | 6.18 ± 2.76* | 4.97 ± 3.41 | 5.81 ± 4.75 |
| Bacteroides | 12.75 ± 7.71 | 3.08 ± 2.35^#^ | 4.57 ± 2.45 | 4.56 ± 1.39 | 2.75 ± 2.10 |
| Helicobacter | 2.79 ± 1.30 | 11.28 ± 7.36^#^ | 6.66 ±6.16 | 7.24 ± 7.29 | 6.32 ± 4.84 |
| KE159538_g | 4.54 ± 4.52 | 7.33 ± 6.23 | 11.43 ± 13.95 | 11.18 ± 15.35 | 4.35 ± 3.99 |
| LLKB_g | 0.62 ± 0.61 | 2.22 ± 0.75^#^ | 3.27 ± 2.07 | 2.06 ± 1.52 | 0.93 ± 1.01* |
| Oscillibacter | 1.61 ± 0.75 | 4.88 ± 0.91^#^ | 3.63 ± 2.39 | 3.22 ± 1.16* | 2.65 ± 1.75* |
| PAC000186_g | 6.47 ± 3.21 | 5.10 ±3.50 | 8.26 ± 5.20 | 5.93 ± 3.87 | 7.17 ± 2.84 |
| PAC000664_g | 2.98 ± 3.42 | 6.07 ± 3.98 | 1.93 ± 1.07* | 3.52 ± 1.94 | 2.03 ± 1.38* |
| PAC001068_g | 7.45 ± 3.14 | 3.97 ±2.30 | 5.29 ± 3.26 | 4.13 ± 1.42 | 12.93 ± 5.69* |
| PAC001091_g | 2.94 ± 2.83 | 4.10 ± 4.09 | 2.10 ± 2.11 | 2.41 ± 2.23 | 3.11 ± 3.15 |
| PAC001228_g | 0.97 ± 0.88 | 3.23 ± 1.86^#^ | 1.00 ± 0.26* | 4.29 ± 5.15 | 1.32 ± 1.33 |
| PAC001485_g | 0.00 ± 0.00 | 0.00 ± 0.00 | 0.02 ± 0.02 | 0.00 ±0.00 | 4.82 ± 4.11* |
| PAC002367_g | 1.23 ± 2.16 | 5.01 ± 8.88 | 0.05 ± 0.06 | 3.73 ± 3.57 | 0.22 ± 0.29 |
| Paraprevotella | 3.53 ± 3.31 | 0.80 ± 0.90 | 0.40 ± 0.28 | 0.34 ± 0.26 | 1.28 ± 1.42 |
| Pseudoflavonifractor | 2.75 ± 0.97 | 5.81 ± 2.63^#^ | 3.20 ± 2.35 | 3.21 ± 1.24 | 2.44 ± 1.38* |

Values indicate means±SD.  ^#^*p*<0.05 vs. NC group. **p*<0.05 vs. IS group.

Table S9. Effects of fecal transplantation of normal mice (FN), orally buspirone-gavaged mice with IS (FBp), and intraperitoneally buspirone-injected mice with IS (FBi) on the gut microbiota composition at the species level in mice with *Escherichia coli* K1 (EC)-induced depression

| Taxon Name | Relative contribution (%) | | | | |
| --- | --- | --- | --- | --- | --- |
|  | NC | IS | IFBp | IFBi | IFN |
| AB599946_s | 5.34 ± 3.04 | 1.16 ± 1.23^#^ | 1.42 ± 0.98 | 2.27 ± 0.83 | 1.10 ± 1.08 |
| Bacteroides acidifaciens group | 3.78 ± 2.65 | 1.23 ± 0.97 | 1.43 ± 0.81 | 0.75 ± 0.55 | 1.05 ± 0.73 |
| EF097112_s | 2.84 ± 1.74 | 1.44 ± 1.18 | 1.63 ±0.73 | 1.46 ± 0.58 | 6.64 ± 2.93* |
| EU505186_s | 0.00 ± 0.00 | 0.00 ± 0.00 | 0.02 ± 0.02 | 0.00 ± 0.00 | 4.69 ± 3.94* |
| EU622763_s group | 7.63 ± 11.22 | 0.28 ± 0.47 | 0.37 ± 0.43 | 0.41 ± 0.52 | 0.04 ± 0.06 |
| EU791023_s | 0.79 ± 1.08 | 0.82 ± 0.81 | 2.60 ± 1.71* | 1.43 ± 1.43 | 2.39 ± 1.26* |
| FJ880724_s | 3.37 ± 3.09 | 0.80 ± 0.90 | 0.40 ± 0.28 | 0.34 ± 0.26 | 1.27 ± 1.40 |
| Helicobacter japonicus | 1.03 ± 0.57 | 2.53 ± 2.09 | 3.42 ± 4.17 | 5.13 ± 5.87 | 1.16 ± 1.17 |
| Helicobacter rodentium group | 1.74 ± 0.83 | 8.70 ± 7.51^#^ | 3.16 ± 2.89 | 2.02 ± 1.95 | 5.09 ± 5.00 |
| KE159538_g_uc | 1.97 ± 4.76 | 2.43 ± 4.95 | 10.57 ± 14.27 | 7.02 ± 16.74 | 1.96 ± 4.44 |
| PAC000198_s | 1.11 ± 1.75 | 0.28 ± 0.32 | 2.53 ± 1.58* | 0.28 ± 0.20 | 0.01 ± 0.01 |
| PAC001065_s group | 3.24 ± 1.66 | 3.26 ± 2.18 | 5.76 ± 3.54 | 4.23 ± 2.67 | 4.98 ± 2.08 |
| PAC001070_s group | 1.31 ± 1.14 | 1.20 ± 1.34 | 2.03 ± 2.50 | 1.03 ± 0.59 | 1.68 ± 2.87 |
| PAC001083_s | 0.56 ± 0.81 | 2.42 ± 2.63 | 0.35 ± 0.42 | 0.71 ± 1.61 | 0.34 ± 0.52 |
| PAC001228_s | 0.15 ± 0.33 | 0.36 ± 0.82 | 0.11 ± 0.22 | 2.45 ± 3.26 | 0.00 ± 0.00 |
| PAC001558_s | 2.40 ± 2.99 | 2.58 ± 4.29 | 1.23 ± 2.23 | 2.18 ± 2.34 | 0.91 ± 1.43 |
| PAC002027_s | 0.67 ± 0.81 | 2.61 ± 2.33 | 0.70 ± 0.31 | 0.34 ± 0.25* | 0.69 ± 0.68 |
| PAC002367_s | 1.23 ± 2.16 | 4.97 ± 8.80 | 0.05 ± 0.06 | 3.68 ± 3.52 | 0.22 ± 0.29 |
| PAC002444_s | 0.99 ± 0.61 | 1.49 ± 0.98 | 2.70 ± 2.01 | 1.93 ± 0.98 | 2.19 ± 3.47 |
| PAC002445_s | 2.83 ± 0.49 | 0.79 ± 0.50^#^ | 1.26 ± 0.73 | 1.21 ± 1.25 | 1.97 ± 1.53 |

Values indicate means±SD.  ^#^*p*<0.05 vs. NC group. **p*<0.05 vs. IS group.

(A)


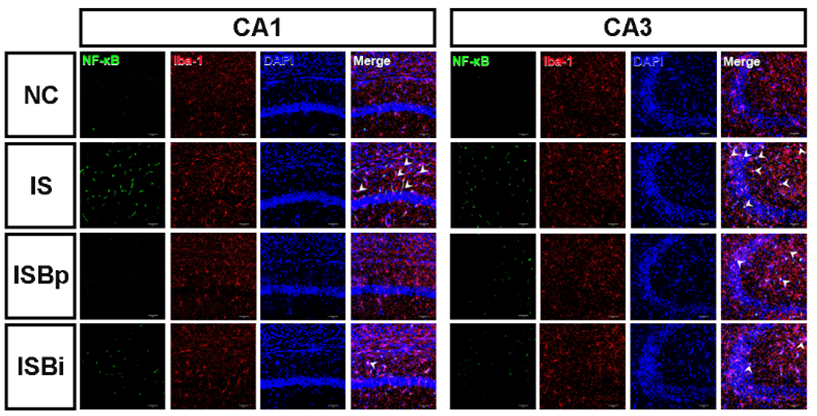


(B)


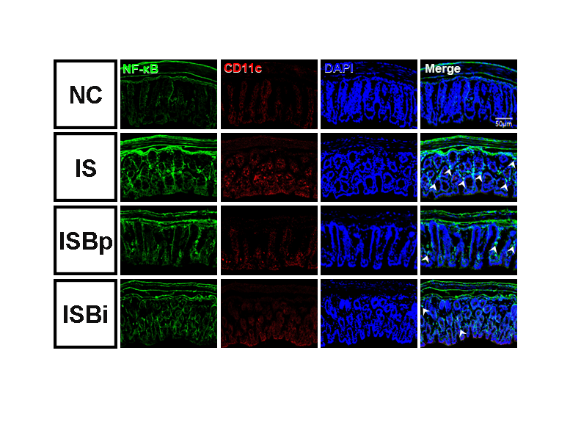


Figure S1. Effects of orally gavage and intraperitoneal injection of buspirone on NF-κB^+^/Iba1^+^ cell population in the hippocampal CA1 and CA3 regions and NF-κB^+^/CD11c^+^ cell population of colon. Buspirone was orally gavaged (IBPp, 5 mg/kg/day) or intraperitoneally injected (IBpi, 1 mg/kg/day) daily for 5 days from the next day after the final exposure to immobilization stress (IS). Normal control mice (NC) was orally treated with vehicle (saline) instead of buspirone.


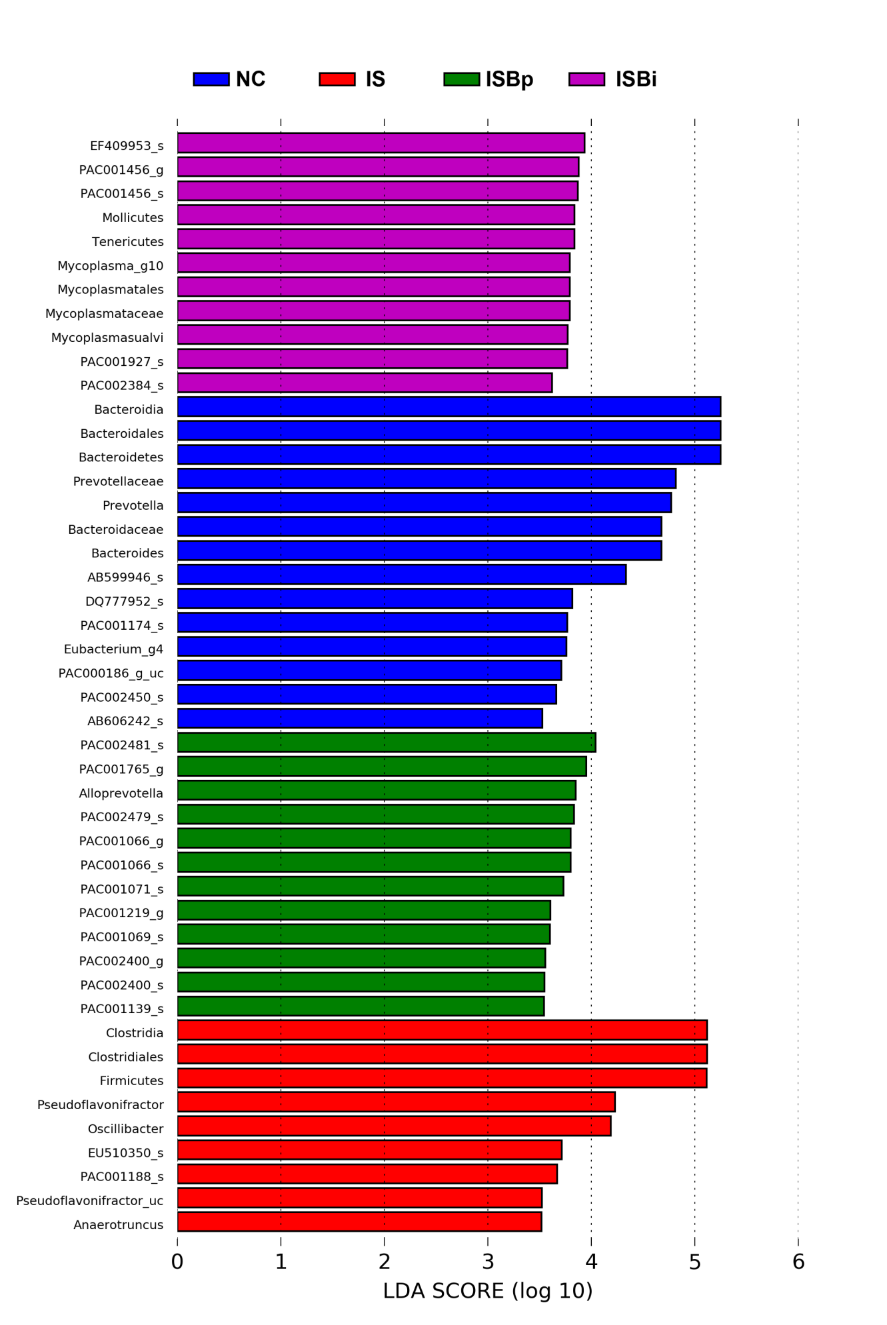


Figure S2. Effect of orally gavaged (ISBp) or intraperitoneal injected buspirone (ISBi) on gut microbiota composition in mice with immobilization stress-induced colitis and gut dysbiosis. Gut microbiota composition was indicated in LDA score. The described strains (in species) were analyzed to the Linear Discriminant Analysis (LDA) along with effect size measurement (LEfSE) in Galaxy (http://huttenhower.sph.harvard.edu/galaxy/). It was used to discriminate significant differentially strains at each taxon level. The threshold logarithmic score set at 3.5 and ranked. Bacterial strains were described based on 16SrRNA sequencing data.





Figure S3. Orally gavage or intraperitoneal injection of buspirone alleviated *Escherichia coli* (EC)-induced anxiety/depression in mice. Effects on EC-induced anxiety/depression-like behaviors in EPM (A, OE) and LDT (B, NT). Buspirone was orally gavaged (EBPp, 5 mg/kg/day) or interaperitoneally injected (EBpi, 1 mg/kg/day) daily for 5 days from the next day after the final exposure to EC. Normal control mice (NC) was orally treated with vehicle (saline) instead of buspirone. Data values were indicated as mean ± SD (n = 6). Same letters are not significantly different (*p* < 0.05).


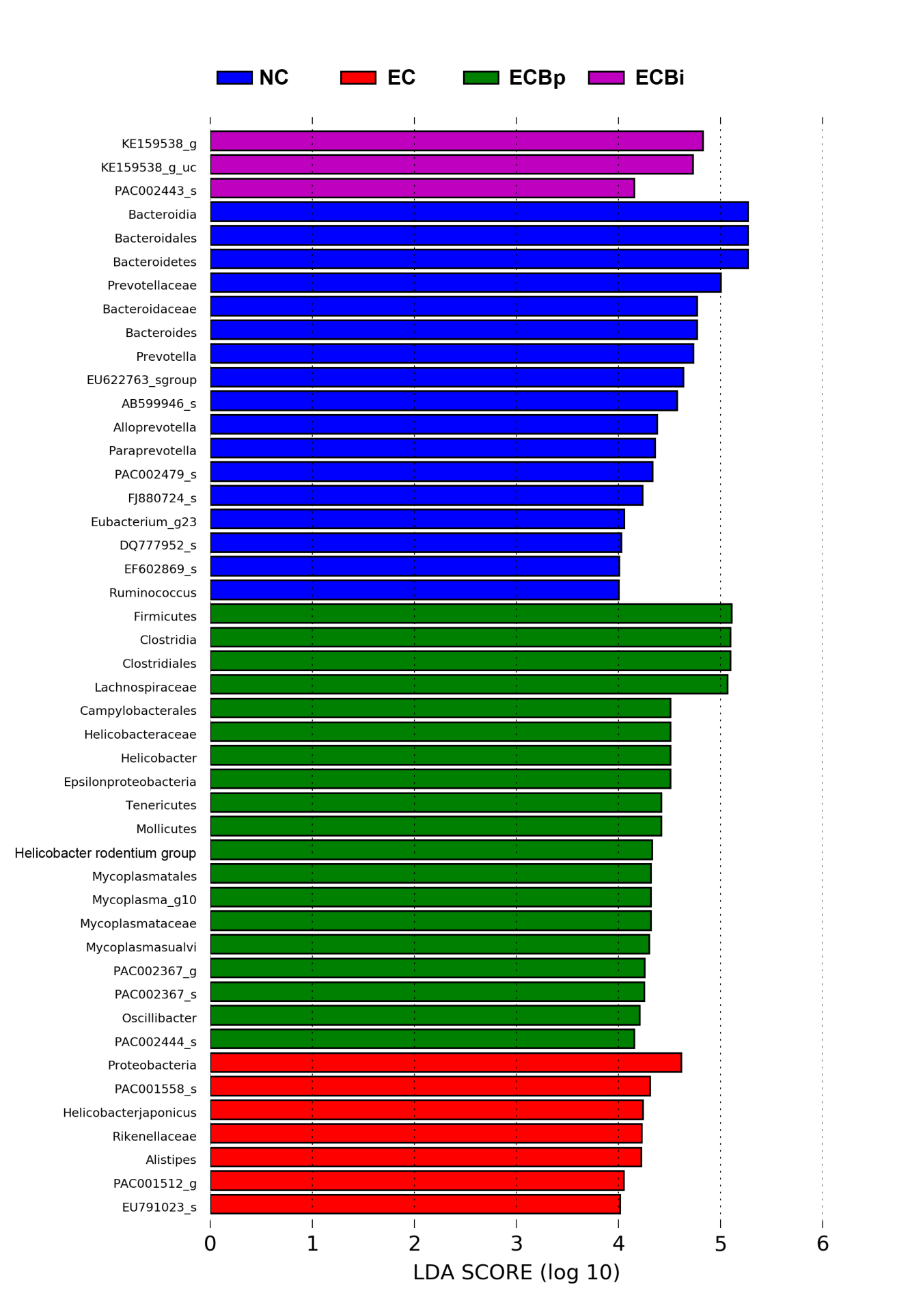


Figure S4. Effect of orally gavaged (ECBp) or intraperitoneal injected buspirone (ECBi) on gut microbiota composition in mice with *Escherichia coli* (EC)-induced colitis and gut dysbiosis. Gut microbiota composition was indicated in LDA score. The described strains (in species) were analyzed to the Linear Discriminant Analysis (LDA) along with effect size measurement (LEfSE) in Galaxy (http://huttenhower.sph.harvard.edu/galaxy/). It was used to discriminate significant differentially strains at each taxon level. The threshold logarithmic score set at 4.0 and ranked. Bacterial strains were described based on 16SrRNA sequencing data.


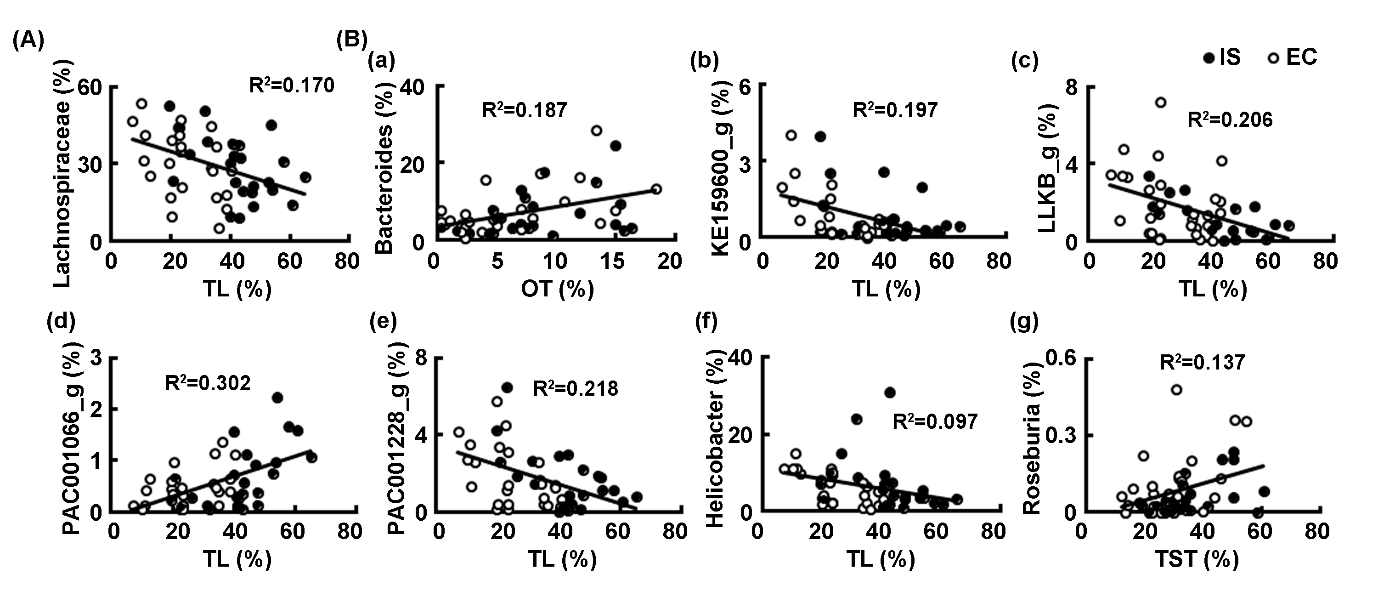


Figure S5. The correlation between anxiety-/depression-like behaviors and gut microbiota in mice treated with and without buspirone. The gut microbiota compositions were analyzed in Family level (A) and genus level (B). The behaviors the times spent in the open arm (OT) in the EPM task, time spent in light box (TL) in the LDT task, and immobility time in the TST were monitored in IS- and EC-exposed mice.


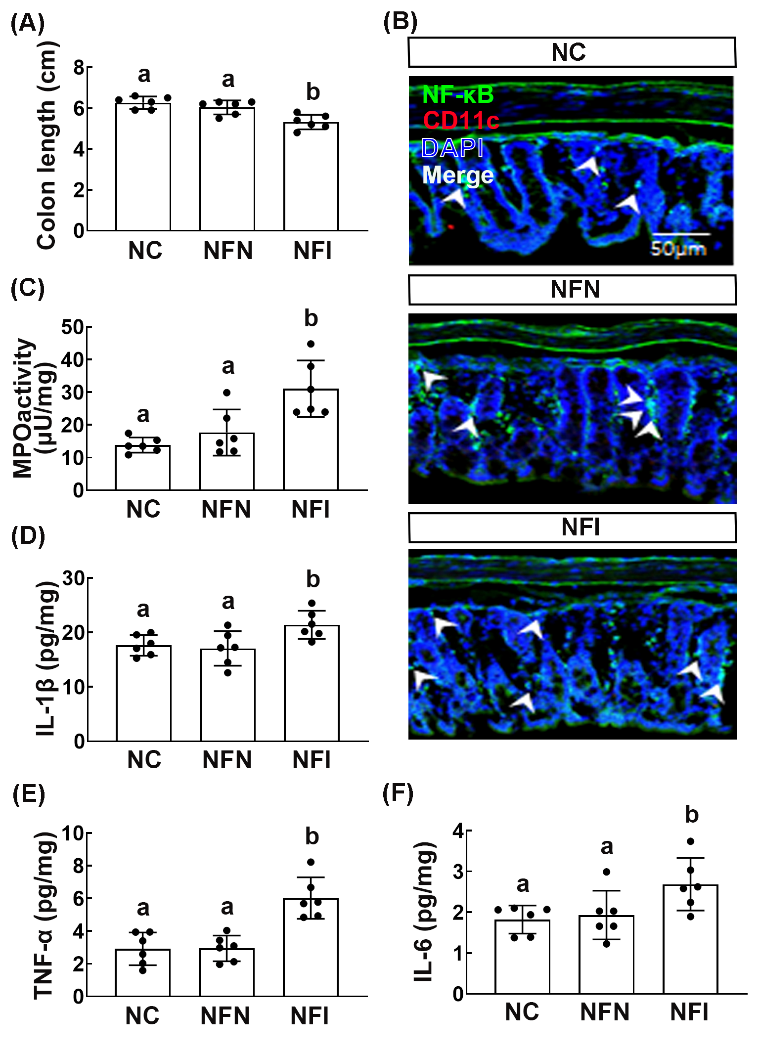


Figure S6. Effects of normal control mouse (NFN) and immobilization stress-treated mouse feces (NFI) transplantation in colon. Effects on NFI induced colon shortening (A), NF-κB+/CD11c+ cell population (B) in colon, myeloperoxidase (MPO) activity (C), IL-1β (D), TNF- α (E) and IL-6 (F) expression. Feces of normal mice (FN) and IS-treated mice (FI) were gavaged daily for 5 days and thereafter treated with saline for 5 days. Data values were indicated as mean± SD (n = 6). Same letters are not significantly different (*p* < 0.05).


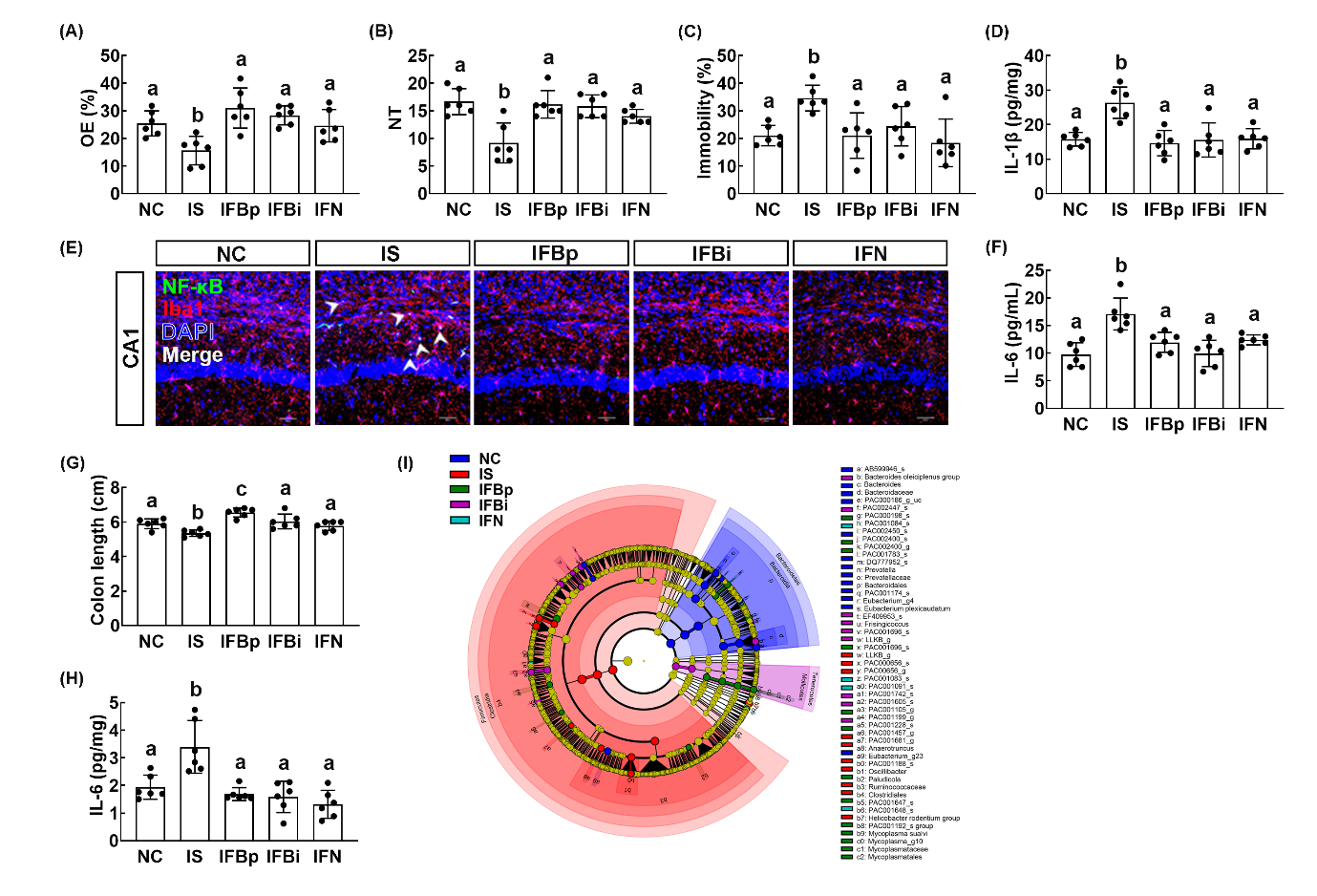


Figure S7. Fecal transplantation of buspirone/immobilization stress (IS)-treated mouse feces or normal control mouse feces alleviated immobilization stress-induced anxiety/depression, colitis, and gut dysbiosis in the transplanted mice. Effects on IS-induced anxiety/depression-like behaviors in EPM (A: open arm enteries [OE]), LDT (B: number of transition into the light box entry [NT]), FST (C). Effects on IL-1β expression (D), NF-κB+/Iba1+ cell population in CA1 region (E) in hippocampus. Effects on IL-6 level (F) in blood. Effects on IS induced colon shortening (A) and IL-6 expression in colon. Cladogram generated by LEfSe analysis in Galaxy (<http://huttenhower.sph.harvard.edu/galaxy/>). It indicates the significant differences in gut microbiota abundances among normal control (NC, blue), IS treated (IS, red), IFBp treated (green), FBI treated (purple), and IFN treated (cyan) groups. Yellow nodes represent species with no significant difference. The threshold logarithmic score set at 4.0 in species level and ranked. Feces of normal mice (IFN), orally buspirone-gavaged mice with IS (IFBp), and intraperitoneally buspirone-injected mice with IS (IFBi) were daily for 5 days from the next day after the final exposure to IS. (I) was created by using LEfSe analysis tool from the galaxy site (<https://huttenhower.sph.harvard.edu/galaxy/>). Normal control mice (NC) was orally treated with vehicle (saline) instead of buspirone. Same letters are not significantly different (*p* < 0.05).

(A)


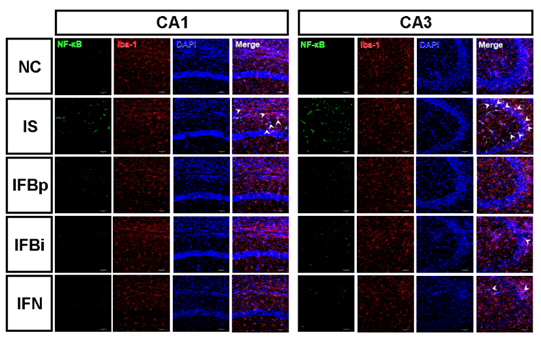


(B)


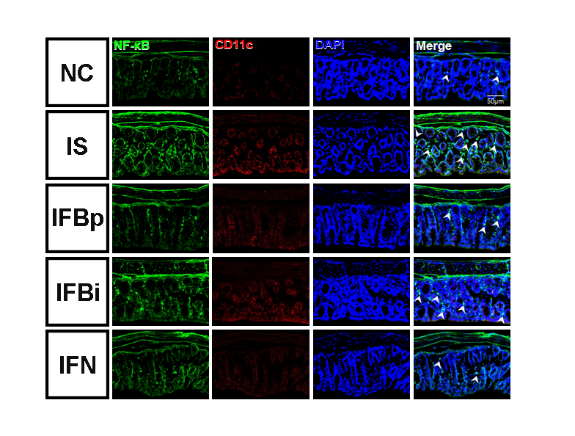


Figure S8. Fecal microbiota transplantation of buspirone/immobilization stress (IS)-treated mouse feces or normal control mouse feces decreased immobilization stress-induced anxiety/depression, colitis in the transplanted mice. (A) Effects on NF-κB^+^/Iba1^+^ cell population in the hippocampus. (B) Effects on NF-κB^+^/CD11c^+^ cell population in the colon. Feces of normal mice (IFN), orally buspirone-gavaged mice with IS (IFBp), and intraperitoneally buspirone-injected mice with IS (IFBi) were daily for 5 days from the next day after the final exposure to IS. Normal control mice (NC) was orally treated with vehicle (saline) instead of buspirone. The present fecal microbiota compositions of NC and IS groups were shared in Figure 2 and Figure S6.


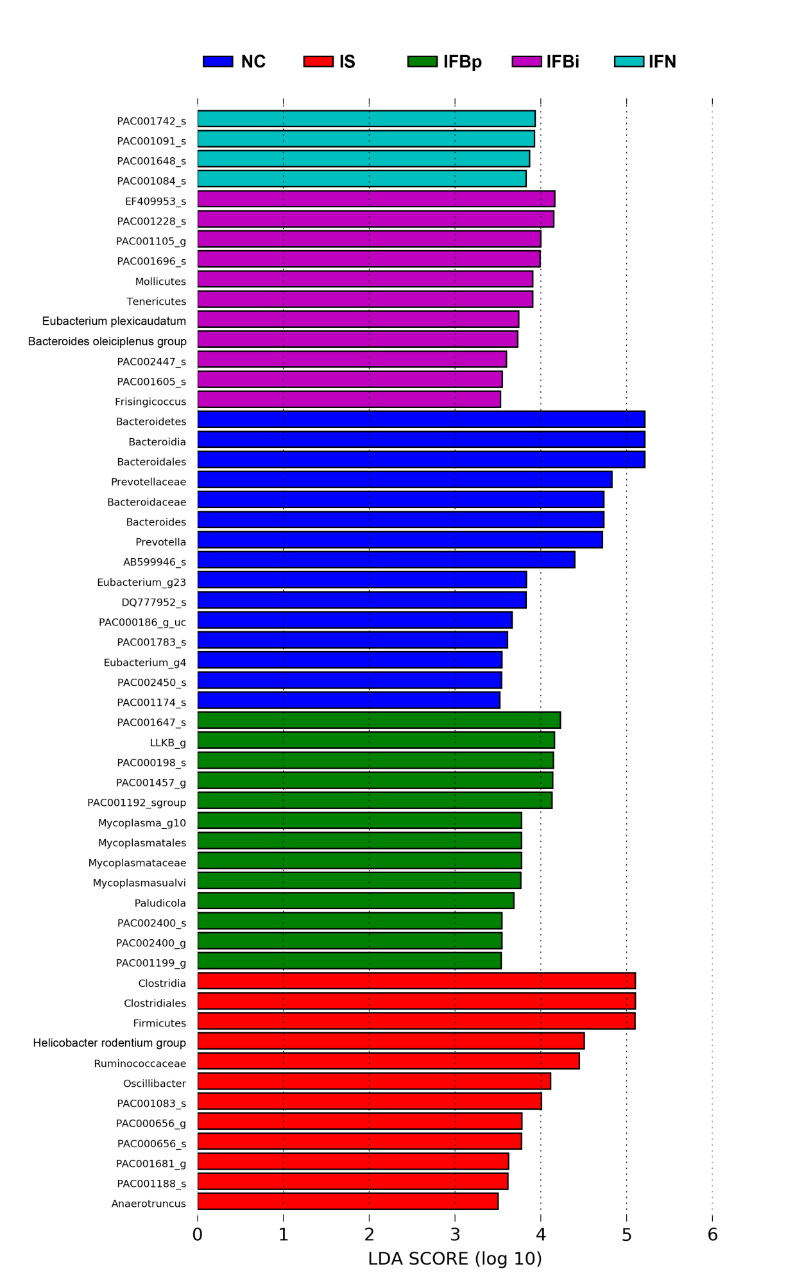


Figure S9. Effect of fecal transplantation of normal mice (IFN), orally buspirone-gavaged mice with IS (IFBp), and intraperitoneally buspirone-injected mice with IS (FBi) on gut microbiota composition in mice with immobilization stress (IS)-induced colitis and gut dysbiosis. Gut microbiota composition was indicated in LDA score. The described strains (in species) were analyzed to the Linear Discriminant Analysis (LDA) along with effect size measurement (LEfSE) in Galaxy (http://huttenhower.sph.harvard.edu/galaxy/). It was used to discriminate significant differentially strains at each taxon level. The threshold logarithmic score set at 3.5 and ranked. Bacterial strains were described based on 16SrRNA sequencing data.
